# Gas Phase Glycerol Valorization over Ceria Nanostructures with Well-Defined Morphologies

**DOI:** 10.1021/acscatal.0c05606

**Published:** 2021-04-06

**Authors:** Louise R. Smith, Mala A. Sainna, Mark Douthwaite, Thomas E. Davies, Nicholas F. Dummer, David J. Willock, David W. Knight, C. Richard A. Catlow, Stuart H. Taylor, Graham J. Hutchings

**Affiliations:** Cardiff Catalysis Institute, School of Chemistry, Cardiff University, Main Building, Park Place, Cardiff CF10 3AT, U.K.

**Keywords:** glycerol, methanol, mechanism, ceria, morphology

## Abstract

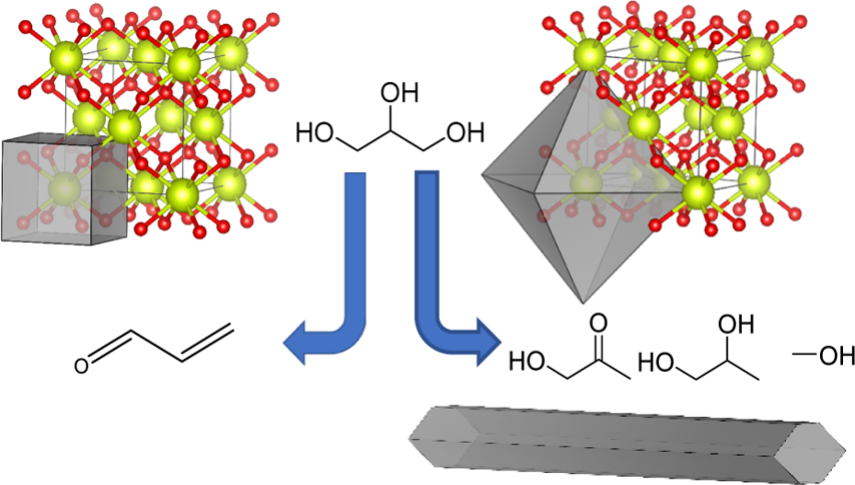

Glycerol solutions were vaporized and reacted over ceria catalysts with different morphologies to investigate the relationship of product distribution to the surface facets exposed, particularly, the yield of bio-renewable methanol. Ceria was prepared with cubic, rodlike, and polyhedral morphologies via hydrothermal synthesis by altering the concentration of the precipitating agent or synthesis temperature. Glycerol conversion was found to be low over the ceria with a cubic morphology, and this was ascribed to both a low surface area and relatively high acidity. Density functional theory calculations also showed that the (100) surface is likely to be hydroxylated under reaction conditions which could limit the availability of basic sites. Methanol space-time-yields over the polyhedral ceria samples were more than four times that for the cubic material at 400 °C, where 201 g of methanol was produced per hour per kilogram of the catalyst. Under comparable glycerol conversions, we show that the rodlike and polyhedral catalysts produce a major intermediate to methanol, hydroxyacetone (HA), with a selectivity of *ca.* 45%, but that over the cubic sample, this was found to be 15%. This equates to a 13-fold increase in the space-time-yield of HA over the polyhedral samples compared to the cubes at 320 °C. The implications of this difference are discussed with respect to the reaction mechanism, suggesting that a different mechanism dominates over the cubic catalysts to that for rodlike and polyhedral catalysts. The strong association between exposed surface facets of ceria to high methanol yields is an important consideration for future catalyst design in this area.

## Introduction

Increased concerns regarding rising CO_2_ levels and the associated environmental consequences have resulted in increased demands for sustainable liquid biofuels. One of the most widely used biofuels is biodiesel with an annual production of *ca.* 30 billion liters in 2014, comprising approximately 1.5% of diesel supplies.^1^ Biodiesel is typically produced through acid- or base-catalyzed transesterification reactions between triglycerides and a simple alcohol, usually methanol, producing fatty acid methyl esters (FAMEs) and glycerol, with the latter accounting for 10% w/w % of the product.^2,3^ While highly pure glycerol is a valuable platform chemical,^4^ with uses in numerous industries, crude glycerol derived from FAME production typically contains high levels of impurities such as water, methanol, unreacted long-chain organic molecules, ash and soap, preventing its use in traditional industrial applications of glycerol, for example, personal care, food, and pharmaceuticals.^4,5^ Accordingly, effective routes for the valorization of crude glycerol are highly desirable to reduce the glycerol surplus and improve the economic viability of biodiesel production.

The valorization of glycerol is not a new concept, with several reviews detailing the progress made in glycerol dehydration,^6,7^ hydrogenolysis,^8−11^ oxidation,^12,13^ gasification,^14,15^ esterification,^16^ etherification,^17^ oligomerization,^18^ acetylation, and carboxylation.^19^ The conversion of glycerol into lower alcohols provides an attractive route for glycerol valorization due to their industrial applicability and potential for fuel blends.^20^ van Ryneveld et al. reported alcohol selectivities exceeding 68% (methanol, ethanol, and propanol combined) over Ni/SiO_2_ catalysts, at a reaction temperature of 320 °C and 60 bar H_2_.^21^ A similar study by Friedrich and co-workers showed that Mo and W catalysts supported on alumina and silica could be used to convert glycerol to lower alcohols,^22^ with a total mono-alcohol (methanol, ethanol, 1-propanol, and 2-propanol) selectivity of >85% at 325 °C and 60 bar H_2_. An ethanol space-time-yield of 1.45 g_ethanol_ kg_cat_^–1^ h^–1^ was reported by Hou and co-workers^23^ over a CoZnO-ZIF-based catalyst, corresponding to an ethanol selectivity of *ca.* 58% obtained at 20 bar H_2_ and 210 °C. Methanol has also been produced from glycerol under supercritical conditions, although harsh reaction conditions were required with both elevated temperatures and pressures (350–475 °C and 250–450 bar).^24^

We have previously demonstrated that under certain reaction conditions, aqueous glycerol solutions can be converted into crude methanol mixtures in the gas phase over simple basic and redox metal oxide catalysts, such as CeO_2_ and MgO, without the need for an external reductant.^25−27^ In our previous studies, we have shown that the reaction conditions strongly influence methanol selectivity, with relatively high reaction temperatures required to achieve high glycerol conversion and methanol yield.

Since Yan and co-workers demonstrated the shape-selective synthesis of ceria nanocrystals, with nanocubes, nanorods, and nanopolyhedra, synthesized by the hydrothermal treatment of Ce(NO_3_)_3_ with NaOH at varying concentrations and temperatures, the effect of the morphology of ceria nanocrystals on their catalytic activity has been the focus of much attention.^41,28−30^ Numerous studies have shown that ceria morphology and surface termination can significantly influence redox,^31^ acid–base,^32^ and defect properties.^33,34^ The effect of the ceria morphology on catalytic activity has been studied for numerous reactions, including examples for which CeO_2_ itself is the catalyst, and those in which CeO_2_ is a catalyst support. In both cases, the ceria morphology has been shown to significantly influence both catalyst activity and selectivity.^35−39^ Pérez-Ramírez and co-workers showed that the (100) surface, predominantly exposed in nanocubes, gives the highest activity for CO oxidation, whereas the (111) surface, which is typically the dominant surface in polyhedral, is the optimal surface for ethylene hydrogenation. Indeed, ceria seems to display this difference in the most active facets for oxidation and hydrogenation reactions quite generally.^40^

We have previously reported on the effect of ceria calcination temperatures and the subsequent physicochemical properties of ceria on the reaction of glycerol, which revealed that there is no clear relationship between the density of defect sites and the reactivity of glycerol or its intermediate products, when samples are compared at a constant space velocity and activity is normalized to catalyst surface area.^26^ This present work examines the effect of the ceria morphology on the conversion of glycerol and subsequent methanol selectivity. As part of this work, reactor space velocities were adjusted to obtain a constant level of glycerol conversion across ceria nanocubes, nanorods, and nanopolyhedra to investigate differences in product distribution with differing morphologies.

## Experimental Section

### Materials

Glycerol (≥99.5%), cerium(III) nitrate hexahydrate (99.9% trace metal basis), and sodium hydroxide (99.8%) were purchased from Sigma-Aldrich. Argon gas was supplied by BOC. All purchased materials were used as received. Deionized (DI) water was provided in-house. Silicon carbide (SiC, ≥ 98%) with a grain size of 300–425 μm was obtained from Alfa Aesar, washed with DI water, and dried prior to use.

### Catalyst Preparation

The three ceria nanostructures were synthesized in accordance with the hydrothermal procedure reported by Yan and co-workers.^41^ For all morphologies, Ce(NO_3_)_3_·6H_2_O (10 mmol) was dissolved in DI water (50 mL). NaOH solution (150 mL) of the appropriate concentration was added to the cerium(III) nitrate solution giving a gel-like precipitate which was stirred at room temperature for 10 min. The suspension (total volume 200 mL) was transferred to a PTFE-lined steel autoclave (total capacity of 300 mL) and heated under autogenous pressure at the appropriate temperature to produce the desired morphology. Once fully cooled, the precipitates were collected by centrifugation, washed thoroughly three times with DI water (3 × 500 mL) and once with ethanol (250 mL), dried *in vacuo* (80 °C; 15 h), and finally calcined in static air at 400 °C for 4 h. The synthesis temperature and concentration of NaOH varied dependent on the desired morphology (rods: [NaOH] = 9 M, *T* = 100 °C; cubes: [NaOH] = 9 M, *T* = 180 °C; polyhedra: [NaOH] = 0.13 M, *T* = 100 °C).

### Catalyst Characterization

Powder X-ray diffraction (XRD) analysis of the catalysts was carried out on a PANalytical X’pert Pro powder diffractometer (Malvern Panalytical, Malvern, UK) using a Cu source operated at 40 keV and 40 mA with a Ge(111) monochromator to select K_α1_ X-rays. Patterns were analyzed from measurements taken over the 2θ angular range 10–80° (step size of 0.016°).

Thermal gravimetric analysis (TGA) and differential thermal analysis were performed using a Setaram Labsys 1600 instrument. Samples (20–50 mg) were loaded into alumina crucibles and heated to 800 °C (5 °C/min) in a flow of synthetic air (50 mL min^–1^). For all specified TGA runs, blank runs were subtracted from the relevant data to remove buoyancy effects.

Brunauer–Emmett–Teller (BET) surface area analysis was performed using a QUADRASORB evo surface area and pore size analyzer. 80 point (40 adsorption and 40 desorption points) analysis was performed using N_2_ as the adsorbate gas at −196 °C. Samples (*ca.* 300 mg) were degassed under vacuum for 3 h at 200 °C prior to analysis.

Transmission electron microscopy (TEM) and scanning TEM were performed on a JEOL JEM-2100 operating at 200 kV. Energy-dispersive X-ray analysis was carried out using an Oxford Instruments X-Max^N^ 80 detector, and the data analyzed using the Aztec software. Samples were prepared by a dry dispersion route and loaded on to 300 mesh copper grids coated with holey carbon films after grinding between glass slides. Particle size analysis was performed by counting 200–250 particles using ImageJ software.

Hydrogen-programmed temperature reduction (TPR) was performed to estimate the reducibility of the catalysts and was performed using a ChemBet chemisorption analyzer (Quantachrome Instruments) equipped with a thermal conductivity detector (TCD). Samples (100 mg) were placed between two plugs of quartz wool in a U-shaped silica tube and pre-treated by heating to 130 °C (15 °C min^–1^) for 1 h under flowing He (80 mL min^–1^). The samples were allowed to cool to room temperature before being heated to 900 °C (10 °C min^–1^) under flowing 5% H_2_/Ar (30 mL min^–1^). Samples were reoxidized by repeating the above procedure under a 10% O_2_/He environment. A second temperature-programmed reduction (TPR) was performed in an identical manner, as the initial analysis, to study any loss of reducibility.

The basicity of the catalysts was investigated by CO_2_ temperature-programmed desorption (TPD). This was performed using a ChemBet chemisorption analyzer (Quantachrome Instruments) equipped with a TCD. Samples (200 mg) were placed between two plugs of quartz wool in a U-shaped silica tube and pretreated by heating to 130 (15 °C min^–1^) for 1 h under flowing He (80 mL min^–1^). CO_2_ was adsorbed at room temperature for a period of 20 min. Physisorbed CO_2_ was removed by heating to 110 °C (15 °C min^–1^) for 1 h under flowing He (80 mL min^–1^). Chemisorbed CO_2_ was desorbed by heating to 900 °C (15 °C min^–1^) for 1 h under flowing He (80 mL min ^–1^); desorbed CO_2_ was monitored by using a TCD (detector current 180 mV; attenuation 1). The same procedure was followed with NH_3_ gas to probe acidic sites, using 10% NH_3_/He as the adsorbate gas. Blank runs were performed without admitting the adsorbate to the sample, resulting in no desorbed species being detected, indicating that the pre-treatment conditions were sufficient to remove adsorbed species and that no catalyst decomposition occurred.

Raman spectroscopy was performed using a Renishaw inVia microscope operated at a wavelength of 514 nm. 10 acquisitions were performed per sample with an exposure time of 10 s; the laser was employed at 1% power.

### Density Functional Theory (DFT) Calculations

All calculations presented here were performed using the Vienna Ab initio Simulation Package (VASP) code^42^ with the core–valence interaction of the electrons represented using the projected augmented wave approach and the valence electronic states expanded in a basis of plane-waves.^43^ A spin-polarized approach with the Perdew–Burke–Ernzerhof (PBE)^44^ functional was employed throughout, and the energy cut-off for the expansion of the plane-wave basis set was set to 550 eV. A Hubbard-*U* term using the Liechtenstein approach^45^ was used to account for self-interaction effects which are particularly significant for the localized Ce(4f) orbitals. We take the value of *U*_eff_ from Loschen et al. who have shown that *U*_eff_(Ce(4f)) = 5 eV gives values for the O 2p–Ce 4f and O 2p–Ce 5d band gaps for bulk CeO_2_ that agree well with experimental estimates.^46^ To account for dispersion effects, the Grimme D3 level^47^ of theory was used.

A convergence criterion for ionic relaxation of 0.01 eV Å^–1^ was used for geometry optimization calculations. The cubic lattice constant was fitted using a Murnaghan equation of state to a series of structures for which atom co-ordinates were optimized at differing fixed cell volumes at a *k*-point grid of 13 × 13 × 13 (Figure S1 and Table S1). The optimal cell constant was found to be 5.469 Å which compares well to the experimental value extrapolated to zero pressure (5.411 Å)^48^ and to the values obtained for the Ce–C, Ce–P, and Ce–R samples in this work (Table 1).

**Table 1:** Structural and Textural Properties of Morphologically Controlled Ceria

sample	morph.^a^	size^a^/nm	exposed planes^a^	(111) peak^b^ degrees	cryst. size^b^/nm	lattice strain^b^/%	lattice param.^b^/nm	surf. area^c^/m^2^ g^–1^	pore volume^c^/cm^3^ g^–1^	ave. pore size^d^/nm
Ce–C	cubes	19.3 ± 2.2	(100)	28.434	20	0.71	0.5432	23	0.159	17.1
Ce–R	rods	90.4 ± 4.6 × 7.1 ± 0.7	(110),(100)	28.490	8	1.72	0.5422	85	0.689	30.1
Ce–P	trun. oct.	10.7 ± 0.9	(111),(100)	28.498	11	1.12	0.5421	65	0.099	5.1

a Measured by high-resolution TEM.

b Calculated from the (111) diffraction peak obtained by XRD, Figure S3.

c Surface area calculated from N_2_ adsorption measurements, Figure S6.

d Calculated from N_2_ desorption isotherm, in accordance with the BJH method. Abbreviations: morph. = morphology, cryst. = crystallite, param. = parameter, surf. = surface, and trun. oct. = truncated octahedra.

For surface calculations, the slab approach was used. Slabs were cut from the optimized bulk CeO_2_ structure, and a vacuum gap of 15 Å was introduced in the direction perpendicular to the slab surface to minimize interaction between images. The most stable surfaces of ceria are (111) and (110).^49^ The (110) Miller planes in CeO_2_ are stoichiometric, while the (111) planes can form stoichiometric stacking units consisting of one Ce^4+^ and two O^2–^ ionic layers, which means that, slab models for the CeO_2_(111) and CeO_2_(110) surfaces can be constructed with zero net dipole across the slab simply by choosing the correct truncation positions for the faces of the slab. In contrast, the (100) surface has a nonzero dipole moment normal to the surface for any choice of truncation and so is a type three surface, according to Tasker’s ionic classification.^49^ To generate the slab model in this case, a slab model of the CeO_2_(100) surface with outer most O^2–^ atomic layers was created and then half of the O^2–^ anions from the top and from the bottom surfaces of the slab were removed to restore stoichiometry and give a slab with no net dipole moment.

The ideal surfaces were modeled by 2 × 2 supercells with seven atomic layers for (100) and (111), while five atomic layers were found to be sufficient to converge the surface energy of the (110) surface. The surface terminations are shown in Figure S2 which shows the pattern of oxygen vacancies used to produce a stoichiometric slab representation of the CeO_2_(100) surface. A *k*-point grid of 3 × 3 × 1 was used for all slab calculations; this choice was made based on the convergence of the surface energy with respect to *k*-point sampling. The upper three layers of the slab models were relaxed during geometry optimizations, and the remaining layers were held fixed at their bulk positions to represent the restraint placed on the surface by the bulk structure.

The calculated surface energies for the optimized surfaces from these slab models were obtained using an approach taking into account the fixed lower layers^50^ (eq S4 in Supporting Information, Section S1). We obtain values of (100): 2.06 J m^–2^ > (110): 1.43 J m^–2^ > (111): 1.14 J m^–2^ (Table S2). While this is the same energetic ordering as reported in the literature using the same PBE functionals^51^ [(100): 1.44 J m^–2^, (110): 1.06 J m^–2^, (111): 0.71 J m^–2^] and found with the PW91 GGA [(100): 1.57 J m^–2^, (110): 1.05 J m^–2^, (111): 0.68 J m^–2^], our values are consistently higher than the earlier work. However, those calculations were carried out without dispersion corrections which would be expected to lead to higher surface energy values since dispersion is an overall attractive energy contribution and atoms at the surface have a reduced number of interactions compared to those in the bulk.

The adsorption energy per adsorbed molecule, *E*_ads_, on these surfaces were calculated from the difference between the calculated total energy of the slab with the adsorbed water molecule, *E*_slab+mol_, and the sum of the energies of the pristine slab, *E*_slab_, and the appropriate number, *n*_mol_, of single water molecules in the gas phase, that is
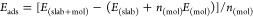
1

A consistent unit cell size and choice of computing parameters were used for both slab and isolated molecule calculations.

To calculate the free energy of hydroxylation for the surfaces, the VASP code was used to evaluate the vibrational modes of the relaxed clean surface, the surface with one monolayer (ML) coverage, and an isolated water molecule. For vibrational calculations of slabs, a single-oxide layer and all adsorbate atoms were included in the degrees of freedom used to form the second derivative matrix. The enthalpy, *H*, entropy, *S*, and free energy, *G*, at a particular temperature, *T*, and pressure, *P*, are then calculated using the formulae
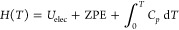
2
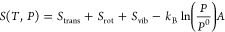
3
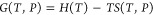
4where *U*_elec_ is the PBE electronic energy of the system calculated by the VASP optimization. The vibrational calculations provide the frequencies for the calculation of the zero-point energy, ZPE, and the heat capacity, *C*_*p*_, and are used to calculate the vibrational contribution to the entropy, *S*_vib_. For the slab calculations, this is the only contribution to the entropy but for the isolated water molecule, the translational and rotational contributions to the entropy, *S*_trans_ and *S*_rot_, are also estimated using standard statistical mechanics approaches. The required partial pressure of water under the experimental reaction conditions was estimated, as described in Supporting Information (Section S4).

For each system, the calculations of the enthalpy and entropy were undertaken using modules from the Atomic Simulation Environment python library.^52^ As part of this work, we have implemented python scripts to read the required data from VASP output files and carry out the set of calculations required to give the enthalpy, *H*, entropy, *S*, and free energy, *G*, changes for the formation of a ML of water from the clean slab and isolated water molecules. The script makes additional checks, such as ensuring that the number of degrees of freedom in reactant and product states is correctly matched.

It is also possible to undertake *ab initio* molecular dynamics (MD) using the VASP code. This facility is used to check the stability of some of the adsorbed configurations using the *NVT* ensemble with Nose thermostat, *T* = 400 K, time step = 1 fs.

### Catalyst Testing

Catalytic reactions were performed using a gas-phase plug flow micro-reactor. Aqueous glycerol solutions (50 wt %) were introduced into a preheater and vaporizer (305 °C) using a high-performance liquid chromatography pump at a flow rates of 0.016 mL min^–1^. The vaporized glycerol feed was swept through the reactor using argon as carrier gas (15 mL min^–1^). All lines were heated to prevent any condensation taking place. Catalysts were pelleted, crushed, and sieved to a uniform particle size (250–425 μm) prior to testing. The catalyst samples (typically 500 mg) were diluted with silicon carbide to a uniform volume (1 mL) and packed into an 8 mm inner diameter stainless steel tube between two plugs of quartz wool. These conditions resulted in mass velocities and space velocities between 1200 and 6000 L h_Ar_^–1^ kg_cat._^–1^ and 2250–9000 L h_Ar_^–1^ L_cat._^–1^, respectively. A thermocouple was placed in the catalyst bed and used to control reaction temperature; reactions were carried out between 320 and 400 °C. Liquid reaction products were collected using an ice-cold stainless-steel trap. A gas bag was attached at the exit line to collect the gaseous products.

Liquid reaction products were analyzed offline using a Varian CP 3800 gas chromatograph (GC1) equipped with a capillary column (ZB-Wax plus, 30 m × 0.53 mm x 1 μm) and an flame ionization detector (FID). Cyclohexanol was used as an external standard. Carbon-based gas reaction products were analyzed offline using a Varian 450-GC gas chromatograph (GC2) equipped with a capillary column (CP-Sil5CB, 50 m × 0.32 mm × 5 μm). Products were detected and quantified by an FID after passing through a methanizer. H_2_ and O_2_ were analyzed using a Varian CP3380 gas chromatograph (GC3) equipped with a Porapak Q column and a TCD. A full list of the identified products and corresponding retention times, according to the GC used, is given in Table S3.

### Reaction Data Interpretation

Equation 5 is used to calculate the glycerol conversion (*C*_GLY_) based on the molar difference between the carbon moles of glycerol fed into the reactor, *g*_mi_, and that detected at the outlet, *g*_mo_
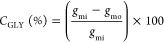
5

The product selectivity [*S*_p_(*x*), carbon mol %] for any product, *x*, was calculated from the moles of carbon recovered of *x* (*x*_C_m__) divided by the total moles of carbon in all detected products, ∑_*x*_*x*_C_m__ (eq 6)
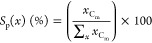
6

The carbon balance is obtained by comparing the moles of carbon accounted for in unreacted glycerol and in all the detected products to the moles of carbon in glycerol entering the reactor
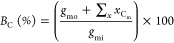
7

Functional group yield (*Y*, carbon mol %) data were calculated from the sum of the selectivity for each product containing that functional group *S*_G_, multiplied by conversion *C*_GLY_, multiplied by the carbon balance *B*_C_, excluding coke (eq 8).
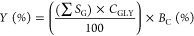
8

The overall carbon balance (*B*_C_tot__) was calculated (eq 9) by dividing the sum of the carbon moles of products (*x*_C_m__), coke (*x*_C_coke__) estimated from post reaction characterization, and unreacted glycerol (*g*_mo_) by the carbon moles of glycerol injected into the reactor (*g*_mi_).
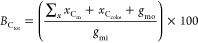
9

The hydrogen balance (*B*_H_) was calculated (eq 10) by dividing the sum of the hydrogen moles of products *x*_H_, hydrogen gas (GC3) (*x*_H_gas__), and moles of hydrogen in unreacted glycerol (*g*_H_mo__) by the moles of hydrogen in glycerol injected into the reactor (*g*_H_mi__).
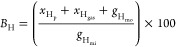
10

The oxygen balance (*B*_O_) was calculated (eq 11) by dividing the sum of the oxygen moles of products (*x*_O_), oxygen gas (GC3) (*x*_O_gas__), and moles of oxygen in unreacted glycerol (*g*_O_mo__) by the moles of oxygen in glycerol injected into the reactor (*g*_O_mi__).
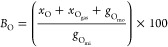
11

The percentage of carbon deposited on the catalyst (coke) was estimated from the mass loss, as analyzed by TGA of the used catalyst. The mass of carbon lost during TGA was converted to the number of moles of carbon retained on the catalyst (*X*_coke_). This was then divided by the carbon moles of glycerol feed over the catalyst (*g*_mi_) (eq 12).
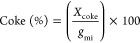
12

The methanol space-time-yield, STY_MEOH_, was calculated (eq 13) from the mass of methanol, *m*_MEOH_, produced per h (reaction time Rt), per mass of the catalyst (*m*_cat_, kg).
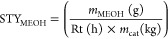
13

## Results and Discussion

### Catalyst Characterization

#### Structural and Textural Properties

Ceria nanostructures with cubic, rodlike, and polyhedral morphologies were prepared in accordance with previously reported procedures, herein referred to as Ce–C, Ce–R, and Ce–P.^41,53^ The main characterization data used to categorize the material structure are summarized in Table 1.

Figure S3 shows the XRD patterns of the prepared materials. All samples were indexed to the cubic fluorite ceria structure (space group *Fm*3̅*m*, JCPDS 01-089-8436), with no impurity or precursor peaks observed. Sharper reflections were observed for Ce–C compared with Ce–R and Ce–P, indicating a higher level of crystallinity for the cubic material than for the rods or polyhedral, which probably arises from the harsher synthesis conditions required to form the cubic morphology, using both concentrated base and a higher reaction temperature of 180 °C. Crystallite sizes, as estimated from the width of the (111) and (200) diffraction peaks (Figure S3), were found to be 21 nm for Ce–C, 8 nm for Ce–R, and 12 nm for Ce–P. Specific surface areas showed an inverse relationship to crystallinity; surface areas calculated in accordance with the BET equation were 35, 85, and 58 m^2^ g^–1^ for Ce–C, Ce–R, and Ce–P, respectively. Calculated lattice parameters were slightly larger than that reported for bulk ceria (0.5411 nm),^54^ with the largest lattice parameter observed for Ce–C (0.5432 nm). An increased lattice parameter can indicate partial reduction of the samples since Ce^3+^ has a larger ionic radius than Ce^4+^ (*r*_Ce_^4+^ = 0.94 Å; *r*_Ce_^3+^ = 1.14 Å). However, the effects on the lattice parameter are complicated by two competing effects—lattice expansions are generally observed with increasing ratios of Ce^3+^/Ce^4+^ due, as noted, to the larger size of Ce^3+^, while lattice contractions can arise with the decreasing crystallite size due to the increased surface/volume ratios for smaller nanocrystals.^54,55^ Calculation of the lattice strain showed Ce–R to have the highest degree of strain, followed by Ce–P and then Ce–C, which is in agreement with a previously published work,^35^ and in agreement with the smaller crystallite size calculated for Ce–R from the XRD pattern.

**Figure 1:**
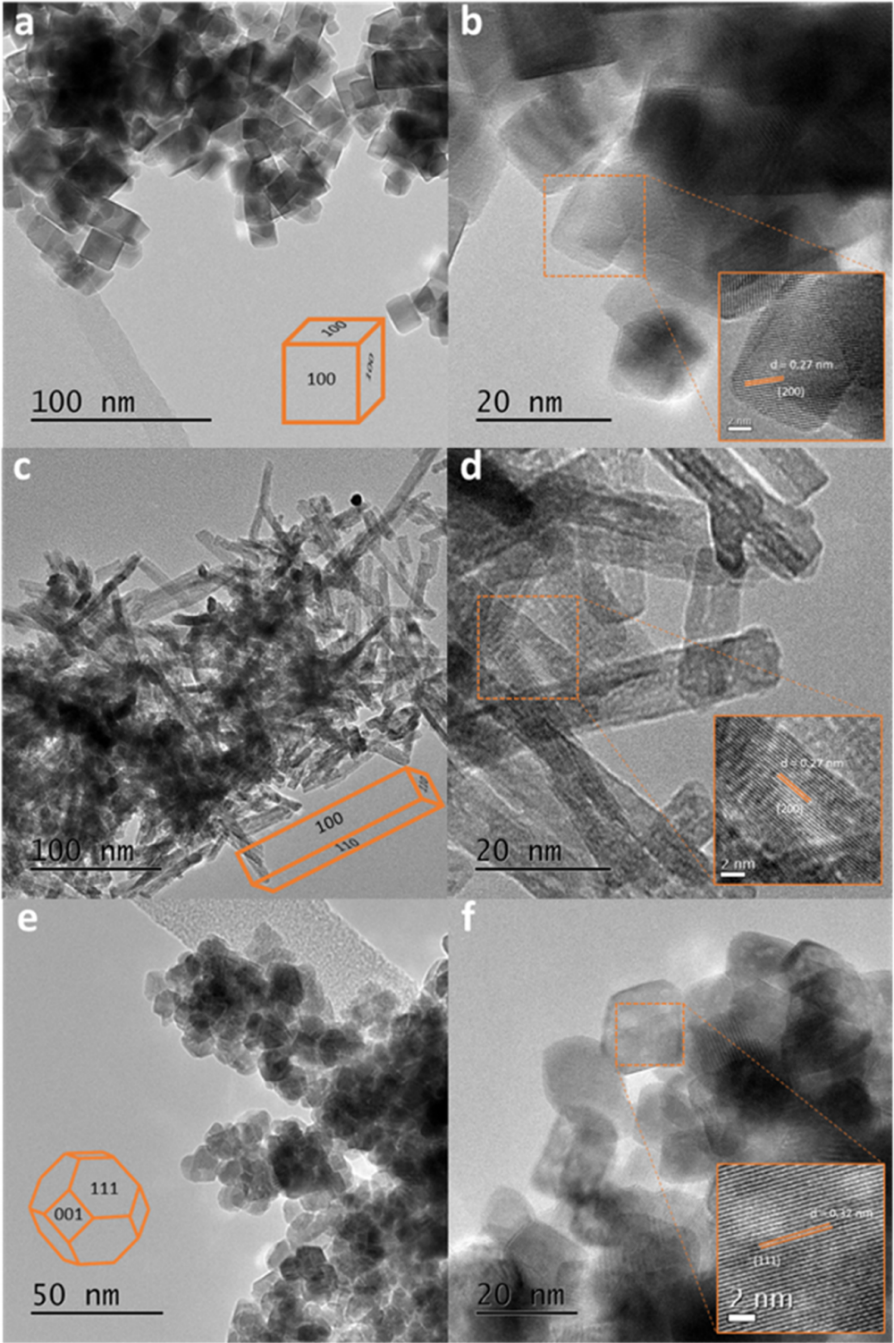
TEM images of ceria Ce–C (a,b), Ce–R (a,b), and Ce–P (e,f).

The morphology of the prepared ceria materials was investigated by TEM; example images are shown in Figure 1. Ce–R and Ce–C have well-defined morphologies with regular rodlike structures expressing (110) and (100) facets and a cubic habit having almost exclusively (100) faces, respectively. A more irregular geometry was observed for Ce–P, which appear to resemble a truncated octahedron morphology most closely with (111) and (100) surface facets. Particle size distributions generated from 200 to 250 individual crystallite images for Ce–P and Ce–C and 150 images for Ce–R are shown in Figure S5. The mean particle sizes, as measured from these TEM estimated distributions, are 19.3 ± 2.2 nm for Ce–C, (90.4 ± 4.6) × (7.1 ± 0.7) nm for Ce–R, and 10.7 ± 0.9 nm for Ce–P, which is in excellent agreement with the crystallite sizes estimated from XRD data.

From the TEM images, interplanar spacings were measured to be 0.27 nm for Ce–C, indicating that the particles are enclosed by (100) facets. Measurements of 0.27 nm were made for Ce–R, in the longitudinal direction. For Ce–P, the dominant lattice spacing was measured to be 0.32 nm, indicating the dominant surface for the polyhedral morphology is the stable (111) surface; additional measurements were made of 0.26 nm, revealing the presence of some (100) facets. Similar findings were made by Trovarelli and co-workers, who demonstrated the exposure of (100) surfaces upon thermally treating octahedral particles, which induce a morphological change to truncated octahedra.^38^ The interplanar spacing measurements were in agreement with numerous previously published studies, reporting the predominant exposure of (100) surfaces in ceria cubes, (110) and (100) surfaces in ceria nanorods, and (111) surfaces in ceria polyhedra.^38,41,53,56,57^

#### Defect Properties and Reducibility

Defects in the materials were probed using visible laser Raman spectroscopy, a technique widely established for the study of ceria-based materials.^58−60^ The main features of Raman spectra obtained are given in Table 2. In the fluorite structure, Ce and O have coordination numbers of 8 and 4, respectively, and the spectra were dominated by a triply degenerate F_2g_ mode at a Raman shift of 462 cm^–1^, corresponding to the symmetrical breathing mode of the Ce–O_8_ local structure.^61,62^ Also present were much weaker bands at wavenumbers of *ca.* 250 and 600 cm^–1^. The latter has been assigned to a defect-induced mode (D), with the relative ratios of the defect band to the F_2g_ band (*I*_D_/*I*_F_2g__) used to estimate the density of defects in ceria-based materials, although the precise origin of this mode has been the focus of much discussion.^59,62−66^

**Table 2:** Defect Properties and Reducibility of Morphologically Controlled Ceria

sample	F_2g_ band^a^/cm^–1^	F_2g_ fwhm^a^/cm^–1^	*I*_D_/*I*_F_2g__^b^	exposed planes^c^	*T*_R_^d^/°C	H_2_ con.^e^/μmol_H_2__ g^–1^	H_2_ con.^e^/μmol_H_2__ m^–2^
Ce–C	463	14.32	0.03	(100)	519	81	3.5
Ce–R	461	37.72	0.07	(110),(100)	491	685	8.1
Ce–P	462	16.26	0.002	(111),(100)	416, 523	572	8.4

a Calculated from Raman analysis.

b The area ratios of the D and F_2g_ band from Raman spectroscopy.

c Identified by TEM.

d The maximum of the low-temperature reduction peak.

e H_2_ consumption calculated from the low-temperature TPR peak (*T* < 620 °C).

Studies by Taniguchi et al. and Luo and co-workers have used visible and UV Raman spectroscopy to probe the defect sites in doped ceria.^59,63^ The band centered around 600 cm^–1^ was ascribed to defects with *O*_*h*_ symmetry whereby the reduced Ce^3+^ cation forms an MO_8_-type complex. However, similar findings were obtained by Luo and co-workers who attributed the band at 600 cm^–1^ to the intrinsic oxygen vacancies required to maintain charge neutrality in the presence of Ce^3+^cations. Wu et al. probed the defect sites of un-doped ceria nanostructures with well-defined crystal planes and proposed an alternative assignment for the defect band at *ca.* 600 cm^–1^.^58^ Their XPS studies showed very similar levels of Ce^3+^ across all morphologies, but in the Raman spectra, nanorods showed the most intense band at 600 cm^–1^ followed by nanocubes with nanopolyhedra showing the least intense defect band. The rods expose (110) and (100) surfaces, cubes expose the (100) surface, and the polyhedra have (111) and (100) surfaces. Wu noted that the intensity of the defect band in the Raman spectra is in agreement with the theoretical energy of defect formation at a surface which is in the order (110) < (100) < (111).^67^ Even though Raman spectroscopy is not a surface-sensitive technique, these results suggest that the surface termination strongly influences the defect sites observed. Consequently, Wu suggested that the band at 600 cm^–1^ is due to oxygen defects which form on the surface and develop into the bulk. The Raman spectra of the as-prepared ceria nanostructures for this study (Figure S7) also showed the *I*_D_/*I*_F2g_ ratio (Table 2) to be in the order Ce–R > Ce–C ≫ Ce–P, indicating that the defect densities in our samples follow the same trend. Additionally, the F_2g_ band observed for the rods was much broader than that for the other nanostructures, consistent with increased defect density and smaller crystallite size, as observed by XRD and TEM.

The reducibility of the ceria samples was assessed using TPR with hydrogen as the reducing gas. Figure S8 shows the H_2_-TPR profiles for each of the CeO_2_ nanostructures with the data summarized in Table 2. It is widely accepted that the high-temperature peak observed during CeO_2_ H_2_-TPR is due to the reduction of bulk oxygen, while low-temperature peaks are attributed to the reduction of surface oxygen species.^68^ For all materials, a high-temperature reduction peak with a maximum at *ca.* 800 °C was observed, which was attributed to the reduction of bulk species. The bulk reduction peak for Ce–R was, however, shifted by *ca.* 40 °C, down to 765 °C. This suggests, similar to the defect densities estimated from Raman spectroscopy, that defect formation in the bulk structure may be influenced by the surface termination. The model for CeO_2_ reduction by H_2_ is proposed to consist of four steps: (1) dissociation of chemisorbed H_2_ to form surface hydroxyl species, (2) Ce^4+^ is reduced to Ce^3+^ upon formation of anionic oxygen vacancies, (3) water desorbs following recombination of surface hydroxyl and hydrogen species, and (4) oxygen vacancies diffuse into the bulk structure.^69^ Since equilibrium is reached between surface and bulk defects through diffusion,^34^ it is plausible that the nature and density of defects in the bulk structure are influenced by surface termination.

At first glance, Ce–C displayed a bimodal peak distribution with a single, broad low-temperature reduction peak with a maximum at 520 °C, in addition to the higher temperature bulk reduction peak. Closer inspection revealed the presence of multiple low-temperature reduction peaks, with additional low intensity peaks observed at 320 and 625 °C. A similar profile was observed for Ce–R, although the main surface reduction peak was shifted to a lower temperature of 478 °C, with a noticeably higher intensity. Additional peaks were detected at 312 and 378 °C, although they were obscured by the main surface reduction peak and appeared as shoulders, preventing peak deconvolution. Well-defined low-temperature reduction peaks were observed for Ce–P, with temperatures centered at 416 and 523 °C. The peak at 416 °C was noticeably sharper than is typically observed, with the lower temperature of reduction, suggesting that the Ce–P nanostructures are more easily reduced than the other morphologies. Surface hydrogen consumption was found to be highest over Ce–R at 685 μmol_H_2__ g^–1^, followed by Ce–P (572 μmol_H_2__ g^–1^), and then Ce–C (81 μmol_H_2__ g^–1^), in agreement with the trend of surface area. However, once normalized to surface area, Ce–P showed the highest H_2_ consumption (8.8 μmol_H_2__ m^–2^) followed by Ce–R (8.1 μmol_H_2__ m^–2^) and Ce–C (3.5 μmol_H_2__ m^–2^), again suggesting enhanced reducibility of Ce–P.

### DFT Results

This work aims to test if the reaction of glycerol over ceria catalysts depends critically on the surface structure of the nanoparticles as the interaction with the high-temperature water/glycerol reaction mixture will depend on the crystal faces present. The synthesis of Ce–C, Ce–R, and Ce–P allows us to compare the reactivity of the major surface facets of these materials. Our DFT calculations focused on the adsorption and reaction of water with the three surfaces identified for the different nanoparticle morphologies in Table 1; (100) relevant to all morphologies, (110) relevant to Ce–R, and (111) relevant to Ce–P, which allows an initial estimate of the relative acidity/basicity of the surfaces and will enable us to estimate the likely level of surface hydroxylation under experimental conditions using *ab initio* thermodynamics.

Plane views of the three surface simulation slabs created for our calculations are shown in Figure S2. Ceria has a fluorite crystal structure. In the bulk unit cell, Ce^4+^ has cubic and O^2–^ tetrahedral coordination. The Ce^4+^···O^2–^ nearest neighbor distance is 2.368 Å in the DFT optimized cubic unit cell compared with the experimental value of 2.343 Å (ICSD structure code 182988).^70,71^ The (110) surface contains exposed sixfold co-ordinated Ce^4+^ cations with a planar arrangement of four oxygen anions around each cation in the surface. Each O^2–^ anion in the (110) surface is three co-ordinate with two surface and one sub-surface Ce^4+^ neighbor. The (111) surface slab is formed by cutting a single Ce^4+^···O^2–^ bond per Ce^4+^ cation; the outer most layer is, again, mainly O^2–^, but seven coordinate Ce^4+^ sites are also available to adsorbates. The process described in the methodology section to create a neutral (100) terminated slab model results in a surface which is still largely oxygen anion terminated but with neutral vacancies which expose additional metal co-ordination sites. Accordingly, each Ce^4+^ that is accessible at the (100) surface has six O^2–^ neighbors and each O^2–^ anion at the surface is bridging between two Ce^4+^ cations.

Water can be adsorbed as a molecule to the surfaces of ceria through interaction of the lone pair density on oxygen with surface Ce^4+^ cations and/or through hydrogen bonding (HOH···O^2–^) to surface anions. It is also possible to adsorb water in a dissociated state to give surface hydroxyl groups by transfer of a proton to a surface anion, with a more basic surface favoring this process. Initially, we considered the adsorption of a single water molecule on the stoichiometric surfaces, calculating the molecular and dissociated adsorption states which, using our simulation slabs, correspond to surface coverages of 0.84 H_2_O nm^–2^ for CeO_2_(100), 0.59 H_2_O nm^–2^ for CeO_2_(110), and 0.42 H_2_O nm^–2^ for CeO_2_(111). In each case, three alternative orientations of the molecule on the surface were explored, and the most negative adsorption energies, calculated using eq 1, are given in Table 3, with the optimized structures, as shown in Figure S9. For the (100) surface, it was difficult to find a starting point for molecular adsorption, as the molecule simply dissociated on optimization of the structure to give a dissociated state with the resulting hydroxyl groups forming a hydrogen bonded pair (Figure S9a). Molecular adsorption was stable on the (110) and (111) surfaces (Figure S9b,c,e) with the adsorption to the (110) surface around 10 kJ mol^–1^ more favorable than to the (111) in good agreement with the earlier results of Parker and co-workers.^51^

**Table 3:** Calculated Adsorption Energies for Water in Molecular and Dissociated States

surface	molecular^a^/kJ mol^–1^	dissociative^a^/kJ mol^–1^	ML^b^/kJ mol^–1^	ML comp.^c^/*n* (*m*/*d*)
(100)		–172	–151	8 (0:8)
(110)	–77	–114	–110	8 (0:8)
(111)	–67	–15	–73	8 (8:0)

a ML = monolayer. Energy calculated for single water molecule in the slab cell.

b Energy per molecule in ML.

c ML composition, *n* = total number of water molecules per simulation cell, *m* = number in a molecular adsorbed state at end of optimization, *d* = number in the dissociated adsorbed state at end of optimization.

Starting structures for the dissociated state for the (110) and (111) surfaces based on the optimized molecular adsorbed state were created by displacing a H atom from water to the nearest surface O^2–^. However, on optimization, these structures consistently recombined to reform the molecular adsorbed state. To obtain the dissociated state quoted in Table 3, the hydrogen atom was placed further away in the supercell, so that the two hydroxyl groups formed do not share a Ce^4+^ neighbor (Figure S9d,f). In this case, the dissociatively adsorbed state on the (111) surface gives an adsorption energy that is actually 52 kJ mol^–1^ less favorable than the molecularly adsorbed case. For the (110) surface, the dissociated state is considerably more stable than the molecular adsorbed form, by 37 kJ mol^–1^. On CeO_2_(110), the dissociated structure has a hydrogen bond for the hydroxyl formed by proton donation to the surface anion to another surface anion with an OH···O distance of 1.82 Å. The other hydroxyl group remains on a Ce^4+^ top site.

So the adsorption of an isolated water molecule highlights the differences in the Lewis basicity of the different surfaces. The (100) surface has the highest affinity for water and adsorbs to a dissociated structure without a reaction barrier. On the (110) surface, water will adsorb as a molecule, but the dissociated form is more stable, so that we expect activated dissociation of the molecule to produce hydroxyl groups on the surface. The (111) surface is the least reactive with dissociation of water energetically disfavored.

To consider higher loadings of water on the surface, slab models for the ML coverage were constructed. A ML of water was taken to consist of one water molecule per exposed Ce^4+^ surface cation and starting structures were constructed with one water molecule placed at each cation site. The surface coverages for the ML structures were 6.69 H_2_O nm^–2^ for CeO_2_(100), 4.73 H_2_O nm^–2^ for CeO_2_(110), and 3.34 H_2_O nm^–2^ for CeO_2_(111). Following on from the low coverage results, dissociated ML structures were then set up for CeO_2_(100) and CeO_2_(110), while molecular adsorption was considered for CeO_2_(111). Table 3 reports the ratio of molecular and dissociatively adsorbed molecules following optimization for each surface at ML coverage along with the calculated adsorption energy per water molecule. The optimized structures for each surface are also shown in Figure S10. As a further check on the stability of the structures produced in this way, a 4 ps MD run was carried out for each surface and 10 structures taken at evenly spaced time points from the resulting trajectory. In all cases, the relaxed structural energy agreed to within 3 kJ mol^–1^ with the original optimized structure.

The ML adsorption energies, as shown in Table 3, follow a similar trend to those for the single water molecule case. The calculated adsorption energy per water molecule shows the strongest binding for the (100) surface, followed by the (110), and the weakest interaction is seen for the (100) case. The CeO_2_(100) surface has the highest density of water molecules adsorbed at the ML coverage: around 40% higher than CeO_2_(110), which results in interactions between the hydroxyl groups which lead to shifts of the oxygen atoms in those groups away from the locations expected from the ceria lattice, as can be seen from the plane view of the optimized surface in Figure S10a. Correspondingly, the adsorption energy per water molecule is some 21 kJ mol^–1^ less favorable than seen in the low coverage calculations. These shifts are not seen in the case of CeO_2_(110) (Figure S10c), and the adsorption energy per water molecule at ML coverage differs by only 4 kJ mol^–1^ from the low coverage value. For a molecularly adsorbed ML on the CeO_2_(111) surface, the adsorption energy per water molecule reported in Table 3 is actually 6 kJ mol^–1^ more favorable than that for a low coverage, and we note in Figure S10e that local networks of hydrogen bonds between water molecules have been formed in this case.

**Figure 2:**
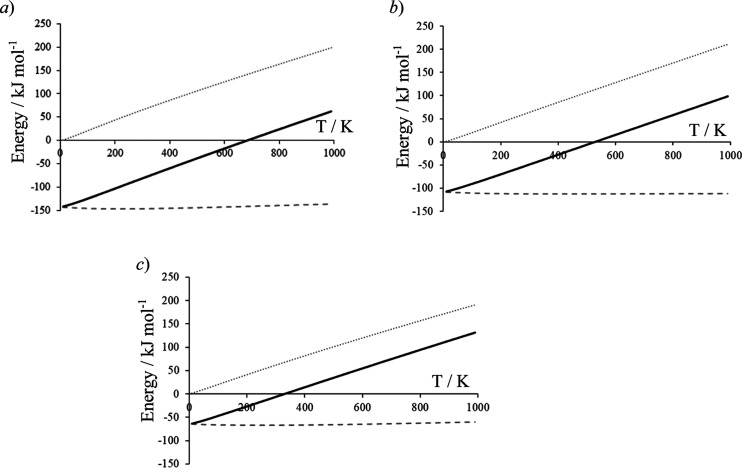
Calculated free-energy change, Δ*G*, for water adsorption on ceria surfaces and the contributions from enthalpy, Δ*H*, and entropy, −*T*Δ*S*, plotted as a function of temperature. Plots are based on the calculated energies for 1 ML coverage with the inclusion of vibrational ZPE and calculated normal modes. Plots are for (a) CeO_2_(100), (b) CeO_2_(110), and (c) CeO_2_(111). For each plot, Δ*G*: solid line, Δ*H*: dashed line, and −*T*Δ*S*: dotted line.

For the ML coverage structures, we have also carried out frequency calculations to allow an estimation of the free energy of adsorption per water molecule for each surface, which are plotted as a function of temperature in Figure 2, at the estimated partial pressure of water under reaction conditions (*P* = 0.60 mbar, see the Supporting Information). As would be expected from the adsorption energies in Table 3, the enthalpy of water ML formation is negative on all surfaces and shows only a very weak temperature dependence. The entropy term in the free energy is also negative as water loses translational and rotational degrees of freedom on adsorption to the surface from the gas phase. The negative entropy contribution means that the free energy increases roughly linearly with temperature. The point at which the free energy crosses the temperature axis gives us an estimate for the temperature up to which water adsorption to the surface to form a ML would be thermodynamically expected. From Figure 2, we estimate this temperature to be 684 K (100), 519 K (110), and 329 K (111). At a higher water partial pressure of 1 bar, Parker et al.^51^ have estimated the temperatures up to which ML coverages would be stable as 825–850 K (100), 575–600 K (110) based on dissociated water, and 325–350 K (111) based on molecular water adsorption energies, which agrees with the trend found here.

In our experimental work, the reaction of glycerol over the ceria catalysts was carried out in the range 593–673 K, meaning that we would expect the (100) surface to be covered in hydroxyl groups from dissociated water under the reaction conditions, while the (110) and (111) surfaces would be largely bare oxide.

#### Acid–Base Properties

The acid–base properties of ceria can vary according to the morphology, which is usually explained by the different coordination states of the cations and anions on the different facets.^32^ TPD of acidic and basic probe molecules is a well-established technique to determine basic and acidic surface sites and can provide information about the strength and density of sites present.^72^ Basic sites were probed by CO_2_ desorption. A blank run was performed without admitting CO_2_ to the sample, which resulted in no CO_2_ being detected, indicating that the pre-treatment conditions were sufficient to remove any adsorbed atmospheric CO_2_. Of the materials tested, Ce–R showed the highest levels of CO_2_ desorption per gram, which is expected since those samples also possess the highest surface area. Ce–C and Ce–P showed very similar quantities of CO_2_ desorbed (Table 4) but with very different desorption profiles (Figure S11). Ce–P showed the most complex distribution of basic sites, with peaks observed at 180, 336, 452, 546, and 786 °C. Using the Redhead approach^73^ (eq S9) (details in Supporting Information, Section S5) as a rough estimate of the corresponding desorption energies for CO_2_ gives values from 130 to 309 kJ mol^–1^, suggesting a wide range of basic sites of different strengths. The peak at *ca.* 452 °C, corresponding to a desorption energy of 210 kJ mol^–1^, was the most intense, suggesting that mainly medium strength basic sites are present with the polyhedral morphology. On the other hand, Ce–C showed a wide desorption profile across the range 125–880 °C, with clear maxima observed at 268 and 736 °C, for which the Redhead equation gives desorption energies of 155 kJ mol^–1^ and 294 kJ mol^–1^, respectively. Deconvolution of the temperature program profile revealed an additional peak at 488 °C. In contrast to the desorption profile of Ce–P, the highest quantity of CO_2_ for Ce–C was desorbed at high temperatures, indicating that a high proportion of strongly basic sites are present. Overall, a higher quantity of desorbed CO_2_ was observed for Ce–R, with a simpler desorption pattern, consisting of two well-defined peaks at 315 and 536 °C, with calculated desorption energies of 169 and 234 kJ mol^–1^. Additionally, the start of a peak can be observed from 850 to 900 °C, indicative of very strong basic sites, although the temperature of 900 °C was insufficient to complete desorption, so this could not be quantified.

**Table 4:** TPD Measurements of Acid–Base Properties for Morphologically Controlled Ceria

morphology	CO_2_ desorbed^a^/μmol g^–12^	CO_2_ desorbed^a^/μmol m^–2^	NH_3_ desorbed^b^/μmol g^–1^	NH_3_ desorbed^b^/μmol m^–2^	basicity/acidity ratio
Ce–C	95	4.1	13	0.56	7.3
Ce–R	138	1.6	23	0.27	6.0
Ce–P	100	1.7	15	0.26	6.5

a Calculated from CO_2_ TPD.

b Calculated from NH_3_ TPD.

The presence and strength of acid sites were probed by NH_3_-TPD. The desorption profiles observed (Figure S12) were very similar to those obtained with a CO_2_ probe, although some minor features were lost. Similar quantities of NH_3_ per gram of the catalyst were desorbed for Ce–C and Ce–P, with higher amounts observed over Ce–R. However, when normalized for surface area, very comparable quantities were desorbed over Ce–R and Ce–P per unit surface area. As described above, the different coordination number of cerium cations and oxygen anions at a surface is dependent on the Miller index of the surface present. It would be expected that surface basicity arising from O^2–^ anions would follow the trend observed in our DFT calculations for the dissociative adsorption of water; (100) > (110) > (111), while the theoretical acidity due to surface Ce^4+^ would follow the trend (100) ≈ (110) > (111).^32^ TPD analysis showed that the basicity followed the theoretical trend, with Ce–C, containing mainly the (100) surface, showing higher levels of basicity than Ce–R and Ce–P, which possessed mainly (110) and (111) facets, respectively. In contrast, acidity measurements deviated from the predicted trend, with Ce–R, containing (110) surfaces, exhibiting lower acidity than theoretically predicted. It should be noted that similar to the CO_2_ desorption, the onset of a high-temperature peak was observed for Ce–R between 850 and 900 °C. This was not observed in the absence of any adsorbate, thus indicating strong interactions between NH_3_ and Ce–R. We note that the DFT calculations suggest that under the pre-treatment conditions used in the TPD experiments, the (111) surfaces would be expected to be cleared of surface water, whereas the (100) and (110) may still have acid sites blocked by hydroxylation. The increased defect density of Ce–R, as measured by Raman spectra (Table 2), could also be responsible for a reduced acidity of cerium cations, resulting in lower Lewis acidity.^32^

#### Glycerol Conversion

An earlier investigation into the influence of some of the physicochemical properties of ceria on glycerol valorization and methanol selectivity showed that surface area, crystallite size, and defect density did not significantly influence the product distribution.^26^ In that work, ceria catalysts were prepared by varying the calcination temperature, resulting in materials with varying surface areas, crystallite size, and defect density. Those reactions were carried out with differing catalyst masses to maintain a constant catalyst surface area, with small alterations made to the carrier gas flow rate to maintain a constant gas hourly space velocity (GHSV). Subsequently, it was found that glycerol conversion was constant with a constant catalyst surface area, and no obvious relationship between ceria crystallite size or defect density, and the conversion of glycerol or intermediate product distribution was observed. As such, it was postulated that the morphology of the catalyst may have more significant effects on product distribution, allowing for enhanced yields of methanol.

**Figure 3:**
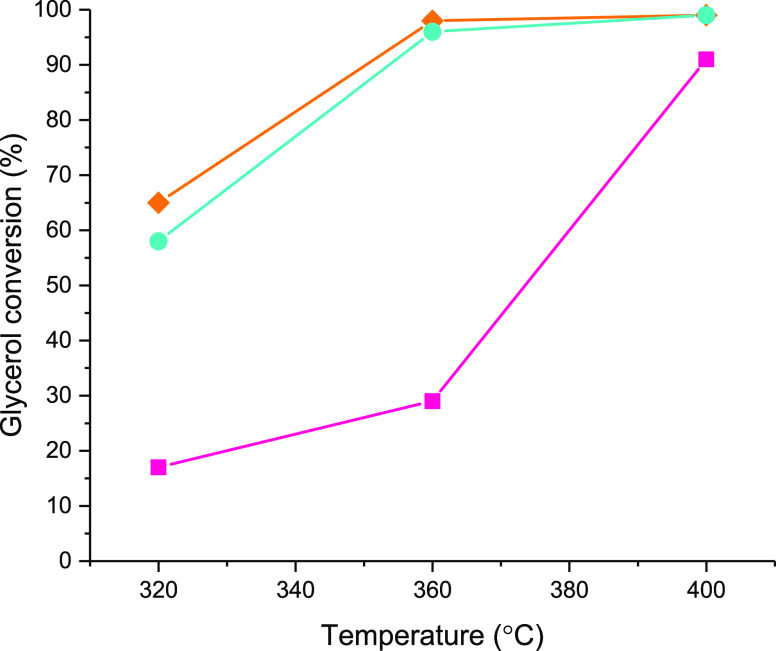
Glycerol conversion at different temperatures at a space velocity of 3600 h^–1^ over Ce–C (pink squares), Ce–R (orange diamonds), and Ce–P (blue circles). Reaction conditions; 50 wt % glycerol (0.016 mL min^–1^), 0.5 g CeO_2_, 15 mL min^–1^ Ar, 3 h, GHSV = 3600 h^–1^.

Initially, in the current work, the catalysts were tested under identical reaction conditions, with catalyst mass, volume, and space velocity kept constant, at reaction temperatures of 320, 360, and 400 °C. Glycerol conversion as a function of temperature is shown in Figure 3. At all reaction temperatures, significantly lower glycerol conversions were obtained over Ce–C than for Ce–R or Ce–P. This was not unexpected, with glycerol conversion following the order Ce–R > Ce–P ≫ Ce–C, which aligns with measured surface areas given in Table 1.

At 320 °C, glycerol conversions of 17, 65, and 58% were obtained over Ce–C, Ce–R, and Ce–P, respectively, with carbon balances of 93, 81, and 91%. A previous work has shown that no significant glycerol conversion occurs at this temperature in the absence of a catalyst. Glycerol conversion was constant across all three materials once normalized to surface area (*ca.* 0.26 mmol_gly_ h^–1^ m_cat_2). Increasing the reaction temperature to 360 °C resulted in significant increases in glycerol conversion over Ce–R and Ce–P, with almost all glycerol converted. In contrast, a modest increase in glycerol conversion was observed over Ce–C, with a conversion of 29% achieved. The carbon balance remained high over Ce–P (>90%) but dropped to 83 and 76% over Ce–C and Ce–R, respectively.

At 400 °C, only traces (<0.1%) of unconverted glycerol were observed over Ce–R and Ce–P after a reaction period of 3 h, while conversion reached 91% over Ce–C. Under these conditions, a significant decrease in carbon balance was observed, at 61, 62, and 67% over Ce–C, Ce–R, and Ce–P, respectively. TGA was used to estimate the coke content of the catalysts (Figure S13). Carbon deposition was highest over Ce–R at 65 mg g^–1^, followed by Ce–C (47 mg g^–1^) and Ce–P (16 mg g^–1^). As shown in Table 5, only a small proportion of carbon lost is in the form of carbon deposition on the catalyst, accounting for <2% of the carbon balance over all materials. Since low levels of carbon deposition were detected, but significant carbon losses were observed, it appears likely that additional products are formed under these conditions which cannot be detected by our typical analysis by GC-FID.

**Table 5:** Glycerol Conversion and Product Distribution over CeO_2_ with Different Morphologies

				mass balance^b^/%			yield^c^/%								
entry	catalyst morphology	reaction *T*/°C	*C*_GLY_^a^/%	C	H	O	Alc.	diols	Ald.	Ket.	Ac.	COx	Unk.	MeOH S.T.Y./g h^–1^ kg_cat_^–1^	carbon deposition^d^/mg g^–1^
1	Ce–C	320	17	93	91	91	1.1	1.9	3.0	2.4	0.7	1.0	5.8	4.01	
2		360	29	83	81	81	2.7	3.5	5.2	5.9	1.1	1.6	4.4	8.97	
3		400	91	61 (62)	53	54	15.6	6.5	5.9	6.4	4.3	7.7	15.5	60.35	47
4	Ce–R	320	65	82	75	73	5.7	8.2	4.1	17.5	5.6	3.3	8.1	39.42	
5		360	98	76	66	63	10.4	9.3	7.8	19.8	8.9	6.3	11.8	90.87	
6		400	>99	62 (64)	48	56	20.2	0.8	7.8	10.8	1.4	15.1	6.5	164.32	66
7	Ce–P	320	58	91	84	82	4.9	7.3	4.3	17.6	4.1	2.9	11.3	40.21	
8		360	96	91	80	76	14.6	12.4	12.4	11.3	11.9	8.9	15.3	121.5	
9		400	>99	67 (67)	51	63	23.5	0.3	3.2	15.0	2.1	18.8	4.3	201.3	16

a Glycerol conversion.

b Carbon mass balance (±3%) of products detected in GC1 and GC2 (values in parenthesis include coke deposited on the catalyst).

c Yield of products detected in GC1 and GC2; Alc., alcohols; Ald., aldehydes; Ket., ketones; Ac., acids; Unk., unknowns.

d Calculated from TGA analysis (Figure S13). Reaction conditions; 50 wt % glycerol/water flow 0.016 mL/min, 0.5 g CeO_2_, 15 mL/min Ar, 3 h.

#### Iso Conversion

We have previously demonstrated a strong relationship between product distribution and glycerol conversion.^26,27^ While appreciable differences in product distribution were observed over Ce–C, compared with Ce–R and Ce–P, the significantly lower glycerol conversion obtained over Ce–C meant that the product distributions could not be directly compared across the three catalysts. In order to overcome this limitation, catalyst masses and subsequent space velocities were altered to achieve comparable levels of glycerol conversion over all catalysts. Catalyst masses were altered to allow flow rates, and therefore partial pressures, to remain constant across experiments.

**Figure 4:**
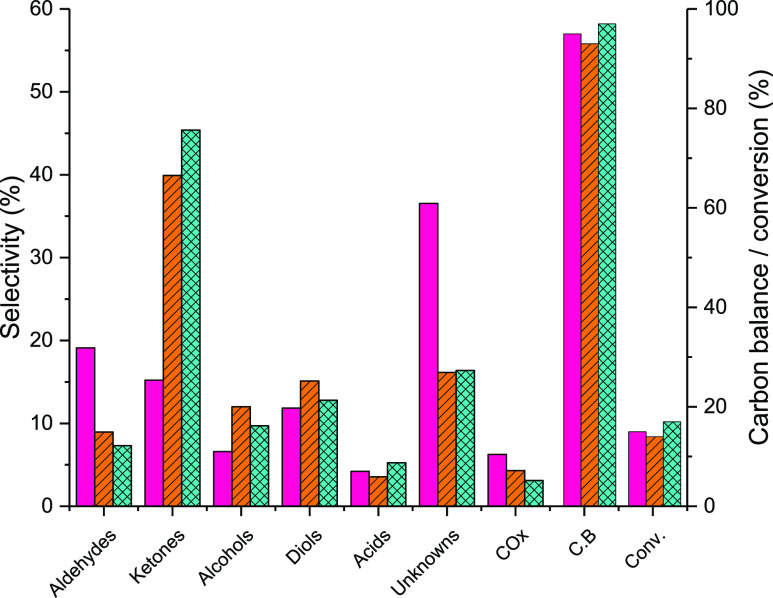
Product group selectivity over Ce–C (pink bars), Ce–R (orange lined bars), and Ce–P (blue hatched bars) where catalyst mass and carrier flow rates were altered to achieve glycerol conversions of ≈15%. Reaction conditions; 320 °C, 50 wt % glycerol (0.016 mL min^–1^), 15 mL min^–1^ Ar, 3 h, GHSV = 3600 h^–1^ (Ce–C), 11,250 h^–1^ (Ce–R), and 9000 h^–1^ (Ce–P).

A glycerol conversion of 17% was attained over Ce–C at 320 °C and a space velocity of 3600 h^–1^. The space velocities were adjusted over Ce–R and Ce–P to 11,250 and 9000 h^–1^, respectively, resulting in conversions of 14 and 16%. At this level of conversion, all three morphologies gave high carbon balance values, >90%, with >95% observed for Ce–R and Ce–P. Due to the diverse range of products formed, products are grouped by their functional groups; product distributions by functional groups at a glycerol conversion of *ca.* 15% are shown in Figure 4 (full product list in Table S6a).

**Scheme 1:**
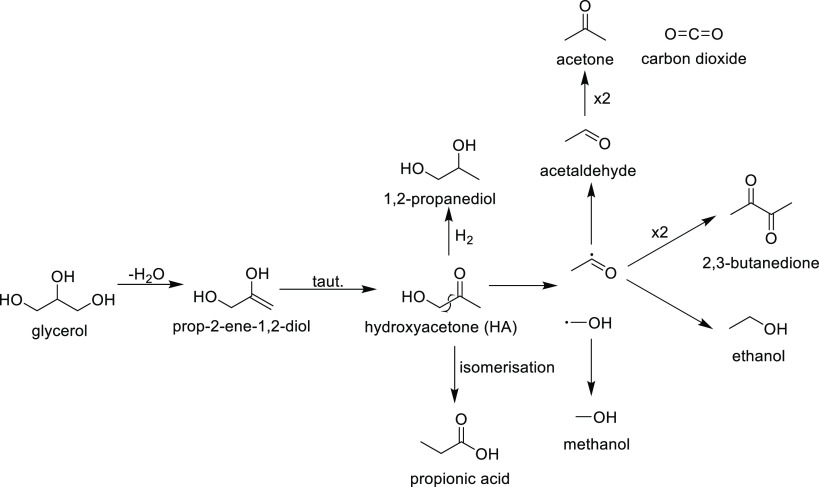
Reaction Pathway Initiated by C1 Dehydration of Glycerol

Product distributions were similar over Ce–R and Ce–P, with hydroxyacetone (HA) as the main product detected, with selectivities of 37 and 44%, respectively, contributing to the high ketone selectivity observed. HA is a glycerol dehydration product, typically formed through the loss of a C1 hydroxyl group in glycerol, which generates an enol intermediate (2,3-dihydroxypropene) that undergoes rapid tautomerization to yield HA (Scheme 1).

Glycerol conversion to HA has been reported in good yields over Lewis acidic materials such as ZrO_2_, Al_2_O_3_, and Nb_2_O_5_^74^ and basic materials such as LaNiO_3_,^75^ La_2_CuO_4_,^76^ and CuO_*x*_–MgF_2_.^77^ The other major products observed over Ce–R and Ce–P were 1,2-propanediol (8 and 5% over Ce–R and Ce–P, respectively), ethylene glycol (7%), methanol (8%), and acetaldehyde (5%), which are secondary products derived from hydroxy acetone, as shown in Scheme 1. The products, as shown in Scheme 1, account for *ca.* 66% of the carbon for reactions carried out with Ce–P and *ca*. 63% when Ce–R is employed. Smaller quantities of numerous other products were found including propanoic acid (*ca.* 2.5%), acrolein (*ca.* 2%), acetic acid (*ca.* 1.5%), 2,3-butanedione (*ca.* 1%), and allyl alcohol (*ca.* 1%), with trace amounts, at less than 1% selectivity of propionaldehyde, acetone, ethanol, propanols, 1,3-propanediol, and CO_2_. Unidentified products were also observed over Ce–R and Ce–P. These products are visible with GC1, but it has not been possible to determine their identity, and selectivity to these unknown compounds is calculated using average response factors from known products with similar retention times.

**Scheme 2:**
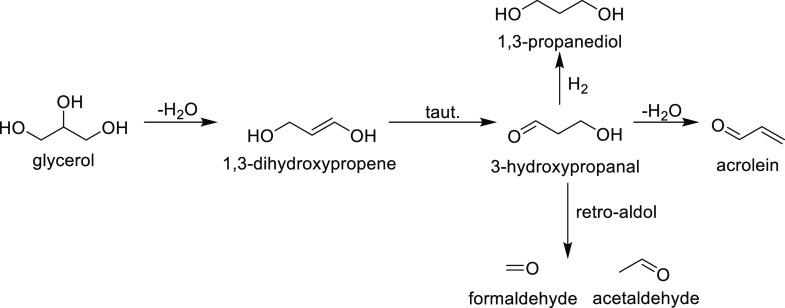
Reaction Pathway Initiated by C2 Dehydration of Glycerol

A significantly different product distribution was found for the reaction of glycerol over Ce–C compared to Ce–R and Ce–P. Over the cubic material, the major product detected was acrolein, with a selectivity of 14%, and a much lower selectivity to HA (14%) was observed compared with Ce–R and Ce–P. Acrolein is a double dehydration product of glycerol, typically initiated by the loss of the C2 hydroxyl group, yielding 3-hydroxypropanal, a highly reactive intermediate which further dehydrates to acrolein (Scheme 2). Some detected products were found to have similar selectivities to those obtained over Ce–R and Ce–P including ethylene glycol (7%), acetaldehyde (4%), propionic acid (3%), and allyl alcohol (1%). However, there are also products, aside from those already discussed, for which there are notable differences; methanol (3%), 1,2-propanediol (2%), 1,3-propanediol (3%), acetic acid (1%), and CH_4_ (5%). In addition, propionaldehyde, acetone, ethanol, 2,3-butanedione, propanoic acid, and CO were observed in small quantities (<1% selectivity). Additionally, a higher selectivity toward unknown products was observed (37%), suggesting that the dehydration of glycerol via the loss of a primary hydroxyl group to yield HA is not the dominant reaction pathway over Ce–C.

Both the major products identified (Ce–R/Ce–P: HA, Ce–C: acrolein) and the selectivities to the minor products (e.g. Ce–R/Ce–P: 1,2-propanediol ≫ 1,3-propanediol and Ce–C 1,3-propanediol > 1,2-propanediol) suggest that over Ce–R/Ce–P, glycerol dehydration at C1 is the more important initial step (Scheme 1), whereas for Ce–C samples, dehydration at C2 (Scheme 2) is dominant. The balance between the two pathways is an important way to control the range of products produced in the reaction and these results suggest that control of a ceria morphology could be an important approach for this to be achieved. We also note that as Ce–C samples are largely CeO_2_(100) terminated, our DFT calculations suggest that the level of hydroxylation for the surface of Ce–C under experimental conditions will be higher than that of the Ce–R/Ce–P morphologies. This would be expected to limit the Lewis basicity of the catalyst and may explain why dehydration at C2 is preferred in this case.

**Figure 5:**
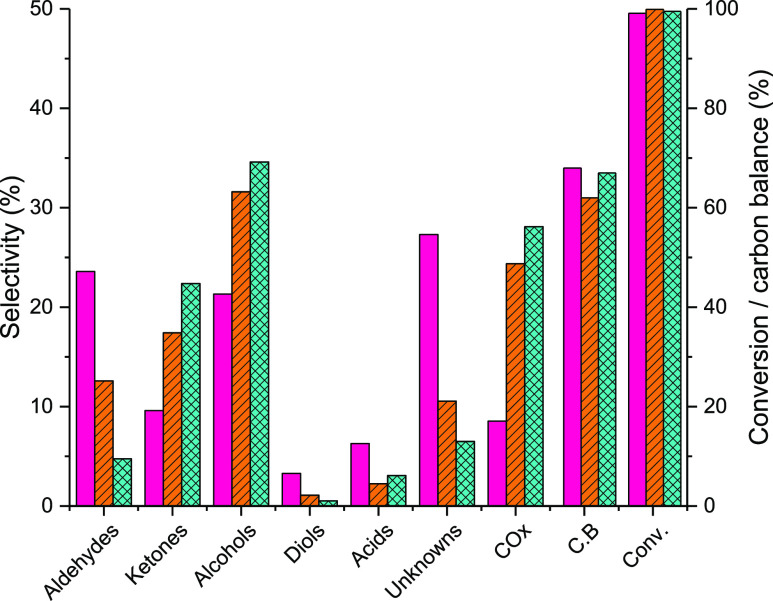
Product distributions at a glycerol conversion of >95% over Ce–C (pink bars), Ce–R (orange lined bars), and Ce–P (blue hatched bars). Reaction conditions; 400 °C, 50 wt % glycerol (0.016 mL min^–1^), 15 mL min^–1^ Ar, 3 h, GHSV = 1800 h^–1^ (Ce–C), 3600 h^–1^ (Ce–R), and 3600 h^–1^ (Ce–P).

As we have previously shown, methanol is a terminal product,^25,27^ so that high methanol selectivity is typically only observed at very high glycerol conversions. To compare methanol selectivity at full glycerol conversion, an increased catalyst mass (and subsequent volume) of Ce–C was used to decrease the space velocity. Since full glycerol conversion was achieved at a space velocity of 3600 h^–1^ over Ce–R and Ce–P at 400 °C, the space velocity was reduced to 1800 h^–1^ over Ce–C to achieve the same level of conversion. Product distributions under these conditions are shown in Figure 5 (full product distribution in Table S6b).

Product distribution by functional group appears to vary somewhat between the three morphologies, although fairly similar product distributions are observed for Ce–R and Ce–P. Alcohol selectivity was in the order Ce–P > Ce–R ≫ Ce–C, with methanol selectivities of 25, 23, and 13%, respectively. Other alcohols included ethanol, 2-propanol, 1-propanol, and allyl alcohol. Under these reaction conditions, only small quantities of HA were observed over all materials, indicative of its role as an intermediate. As shown in Scheme 1, HA undergoes a radical fragmentation process, related to a Norrish type 1 reaction, generating methanol and acetyl radicals. The methanol radical is reduced to yield methanol, whereas the acetyl radical is reduced to acetaldehyde, with further reduction to ethanol possible. Alternatively, the coupling of two acetyl radicals generates 2,3-butanedione; the formation of 2,3-butanedione, a C_4_ product, from glycerol, a C_3_ molecule, provides good evidence for the fragmentation of HA and the presence of acetyl radicals. Higher aldehyde selectivity was observed over Ce–C (24%) compared to the other morphologies, the main aldehyde produced was acetaldehyde (16%) with smaller quantities of propionaldehyde and acrolein also detected. Acetaldehyde is a product which can be formed through either HA fragmentation (Scheme 1) or via a retro-aldol mechanism (Scheme 2).

The selectivity to acrolein over Ce–C was significantly lower than that was observed at low glycerol conversions, likely due to the higher reaction temperature used favoring the retro-aldol fragmentation of the 3-hydroxypropanol intermediate, as previously reported by other authors.^78^ A total aldehyde selectivity of 13% was observed over Ce–R, composed of acetaldehyde (6%), propionaldehyde (6%) along with small quantities of acrolein and butyraldehyde. Less than 5% of the total product distribution over Ce–P consisted of aldehydes, primarily due to the low acetaldehyde selectivity observed. The low aldehyde selectivity over Ce–P, and to a lesser extent Ce–R, was attributed to ketonization reactions, which have been widely reported over CeO_2_ and CeO_2_-containing catalysts,^79−81^ here two acetaldehyde molecules and one water molecule react to give acetone, carbon dioxide, and two equivalents of hydrogen. No H_2_ gas was detected throughout these experiments, but hydrogen is required to form several products, including methanol, suggesting that it was consumed *in situ*. A selectivity of around 20% toward ketones was observed over Ce–R and Ce–P, despite the high levels of HA conversion. This was attributed mainly to acetone and 2,3-butanedione, with small quantities of HA, cyclopentanone, and hexanone also detected. As shown in Figure 5, at complete glycerol conversion, high ketone selectivity is typically accompanied by low aldehyde selectivity and high COx selectivity. Both CO and CO_2_ were observed over all materials; these are undesirable byproducts which are typically produced under conditions yielding high levels of methanol. Future work will focus on reducing COx levels without reducing methanol selectivity.

At full glycerol conversion, the carbon balances (excluding catalyst coking) over all three catalysts were low at 63, 62, and 67% over Ce–C, Ce–R, and Ce–P, respectively. The amount of carbon deposited on the catalysts was estimated by TGA. Taking catalyst coking into account, very modest increases in carbon balances were observed, taking the total carbon balances to 64, 64, and 68% over Ce–C, Ce–R, and Ce–P, respectively. Total organic carbon analysis was performed on the liquid phase products to determine the total carbon balances (Table S4). It was found that for all catalysts, the overall carbon balances were *ca.* 95%, with the remaining 5% attributed to reactor fouling. The discrepancies between observed carbon balances, as calculated from GC analysis, and total organic carbon analysis can be attributed to the formation of insoluble humin-type products, which were visibly present; similar findings were made by Hernandez et al. for the reaction of glycerol over lanthanum-based catalysts.^75^

**Figure 6:**
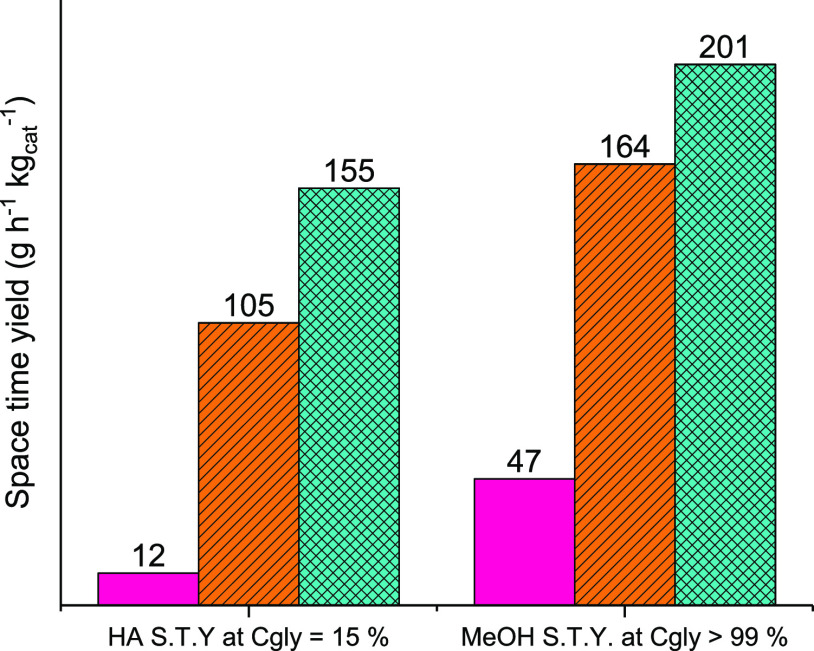
HA and methanol space-time-yields over Ce–C (pink), Ce–R (orange lined), and Ce–P (blue), where catalyst mass and carrier flow rates were altered to achieve glycerol conversions of *ca.* 15 and >99%. Low conversion reactions performed at 320 °C; high conversion reactions performed at 400 °C and GHSVs between 1800 and 11,250 h^–1^.

At complete glycerol conversion, methanol space-time-yield (S.T.Y.) follows the order of methanol selectivity, with the highest space-time-yield obtained over Ce–P (201 g h^–1^ kg_cat_^–1^), followed by Ce–R (164 g h^–1^ kg_cat_^–1^) and then Ce–C (47 g^–1^ h^–1^ kg_cat_^–1^). This follows the same trend as HA space-time-yield at low conversion, as shown in Figure 6. The difference in the reaction mechanism between morphologies, with Ce–R/Ce–P favoring C1 dehydration (Scheme 1), while over Ce–C dehydration at C2 (Scheme 2) is observed, results in significantly lower methanol production achieved over Ce–C materials as methanol is not generated through this route. The high HA S.T.Y. achieved over Ce-R/Ce–P at low glycerol conversion is indicative that Scheme 1 is a major reaction pathway for these catalysts, and as HA is one of the major intermediates in methanol formation, there is a correspondingly high methanol selectivity and space-time-yield at high glycerol conversion.

## Conclusions

Aqueous glycerol solutions were reacted as vapor phase reagents over three types of ceria catalysts that were prepared with cubic, rodlike, and polyhedral morphologies. The yield of major products such as HA at low conversion and methanol at high conversion has been discussed with respect to the surface properties of the three ceria catalysts. We proposed that the density of exposed surface facets is strongly linked to the acid/base properties of the catalysts in addition to the degree of surface hydroxylation and the defect density. DFT calculations suggest that there will be significant differences in the degree of surface hydroxylation, with the morphologies exposing (100) surfaces being hydroxylated at reaction temperatures, unlike those exposing (110) and (111) surfaces. Reactions conducted at iso-conversion indicated that over the rods and polyhedral ceria, the initial product is 2,3-dihydroxypropene, and over the cubes, the product is 1,3-dihydroxypropene. This difference in the reaction mechanism results in a high space-time-yield of HA over the rods and polyhedral catalysts at low conversion. HA is one of the major intermediates to methanol formation, and as such, the methanol space-time-yield over the polyhedral was found to be >4 times that over the cubes. The strong dependence of the product yields to the density of surface facets and the corresponding surface properties should be considered for future work in this area.

## Supporting Information Available

Catalyst characterization, DFT calculations, analytical techniques for evaluation of catalytic performance, full product distributions obtained, and comparison of catalytic systems (PDF)
