# Supplementary material for: Gas Phase Glycerol Valorization over Ceria Nanostructures with Well-Defined Morphologies
Source: ACS Catal. 2021 Apr 6;11(8):4893–907. doi: 10.1021/acscatal.0c05606 (PMC8154328; doi:10.1021/acscatal.0c05606)
Supplement: Supplementary file 1 — cs0c05606_si_001.pdf [file cs0c05606_si_001.pdf]

Supporting Information

Gas Phase Glycerol Valorisation over Ceria Nanostructures with Well Defined Morphologies

Louise Smith, Mala A. Sainna, Mark Douthwaite, Thomas E. Davies, Nicholas F. Dummer,* David J. Willock, David W. Knight, C. Richard A. Catlow, Stuart H. Taylor and Graham J. Hutchings*.

Cardiff Catalysis Institute, School of Chemistry, Cardiff University, Main Building, Park Place, Cardiff, CF10 3AT, UK.

*Corresponding authors: dummernf@cardiff.ac.uk ; hutch@cardiff.ac.uk

Page	Description
S3	Figure S1. The effect of cell volume on the calculated lattice energy for a series of k-point sampling grids.
S4	Table S1. Structural parameters for the cubic cell of bulk ceria refined using the Murnaghan approach.
S5	Table S2. Calculated surface energies from slab model simulations.
S6	Figure S2. Plan views of the surfaces of slab models used for the simulations
S7	Fig S3. PXRD patterns of the catalysts
S8	Fig S4. TEM images showing the d-spacing and the corresponding FFT patterns
S9	Fig S5a-c. Particle size distributions from TEM images
S10	Fig S6a-c. N_2 sorption isotherms
S11	Fig S7. Visible Raman spectra of the materials
S12	Fig S8. H_2-TPR profiles
S13	Fig S9. Structures for H_2O on ceria surfaces
S14	Fig S10. Optimised structures for 1 ML coverage of H_2O
S15	Thermochemistry calculation details and Estimation of partial pressures
S16	Fig S11 & fig S12. CO_2 and NH_3 TPD profiles
S18	Estimation of CO_2 desorption energy using the Redhead equation.
S19	Table S3. GC retention times for full product list
S20	Table S4. Qualitative LC-MS analysis conditions

S21	Table S5a. Full product distribution at a space velocity of 3600 h^{-1} and reaction temperature of 320 °C
S22	Table S5b. Full product distribution at a space velocity of 3600 h^{-1} and reaction temperature of 360 °C
S23	Table S5c. Full product distribution at a space velocity of 3600 h^{-1} and reaction temperature of 400 °C
S24	Table S6a. Full product distribution at *iso*-conversion at C_{gly} ≈ 15% and a reaction temperature of 320 °C. Space velocities: Ce-C = 3600 h^{-1}, Ce-R = 11250 h^{-1}, Ce-P = 9000 h^{-1}.
S25	Table S6b. Full product distribution at *iso*-conversion at C_{gly} ≈ 99% and a reaction temperature of 320 °C. Space velocities: Ce-C = 1800 h^{-1}, Ce-R = 3600 h^{-1}, Ce-P = 3600 h^{-1}.
S26	Table S7. Full carbon based product distribution at *iso*-conversion at C_{gly} ≈ 99% and a reaction temperature of 320 °C. Space velocities: Ce-C = 1800 h^{-1}, Ce-R = 3600 h^{-1}, Ce-P = 3600 h^{-1}<br><br>Figure S13. Post reaction TGA profiles
S27	Table S8. Glycerol conversion and main product selectivies as a function of contact time at various temperatures
S28	Table S9. Glycerol conversion to MeOH over various catalysts
S29	References

S1 DFT Calculation Parameter Choices

The cell vector for each *k*-point sampling grid was obtained by optimisation of atom co-ordinates at a series of cell volumes by systematically expanding and contracting the cell vector around the experimentally reported value. The resulting energy vs cell volume plots for expansion factors up to ±1% in 0.1% steps are shown in Figure S8.

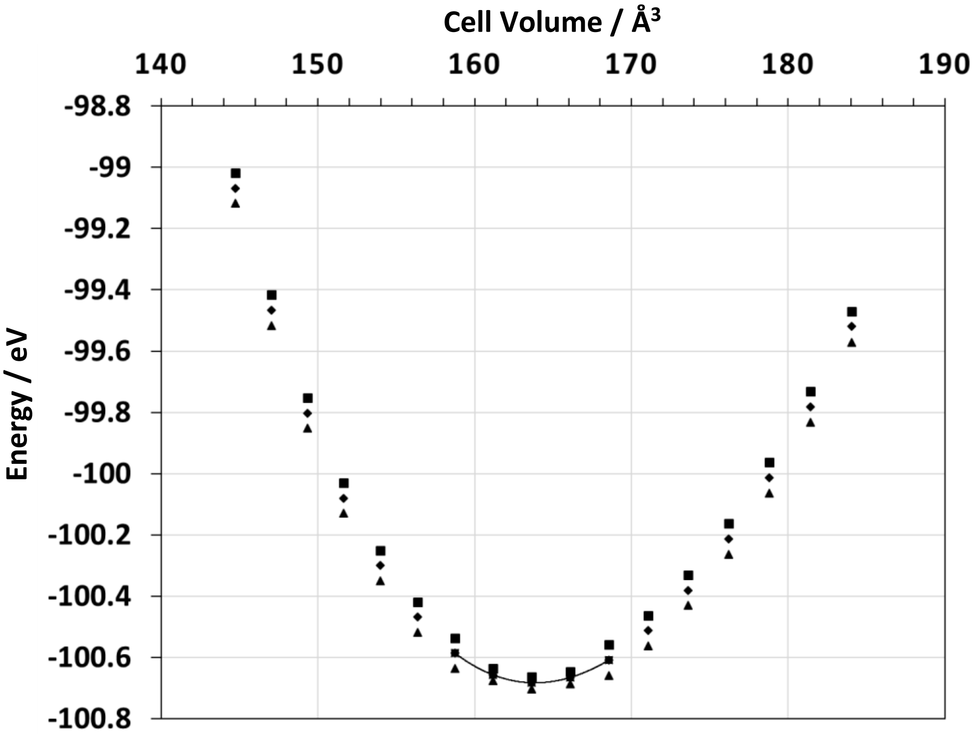

Figure S1: The effect of cell volume on the calculated lattice energy for a series of k-point sampling grids. Plot shows result of k-point sampling of 3x3x3 (diamonds), 5x5x5 (squares), and 13x13x13 (triangles).

From the region close to the minimum (indicated by the lines of best fit on figure S8) an initial estimate for the bulk modulus can be made based on a quadratic fit:

$$K_0 = V_0 \frac{\partial^2 E}{\partial V^2} = 2aV_0 \qquad \text{(S1)}$$

where V_0 the cell volume at the minimum cell energy, $E(V_0)$ and a is the coefficient for the V^2 term in the quadratic fit.

The cell energy as a function of cell volume, $E(V)$, can be fitted over a broader range using the Murnaghan equation of state:

$$E(V) = E_0 + \frac{K_0 V}{K_0'}\left(\frac{(V_0/V)^{K_0'}}{K_0'-1} + 1\right) - \frac{K_0 V}{K_0'-1} \qquad \text{(S2)}$$

where the additional parameter, K_0' is the derivative of the bulk modulus, K_0, with respect to pressure:

$$K_0' = \left(\frac{\partial K}{\partial p}\right)_T \quad \text{(S3)}$$

The data from the cell expansion calculations in VASP was fitted to the Murnaghan equation of state using a simple minimisation of the sum of squared deviations between data and values calculated from the equation of state with V_0, E_0, K_0 and K_0' as variable parameters using the Excel spreadsheet solver function. The initial values of the first 3 parameters were taken from the quadratic fit results and the initial value of K_0' was set to 4. The final values obtained by the fitting procedure are given in Table S1.

Table S1. Structural parameters for the cubic cell of bulk ceria refined using the Murnaghan approach.

k-point sampling level	V_0 / Å^3	E_0 / eV	K_0 / eV Å^{-3}	K_0'
3×3×3	163.85	-100.683	1.150	4.502
5×5×5	163.85	-100.683	1.150	4.497
13x13x13	163.83	-100.683	1.151	4.563

All structural parameters for the cubic cell are converged at a k-point sampling level of 5×5×5. The calculated cell volume of 163.85 Å^3 corresponds to a cell vector of 5.469 Å, in good agreement with the experimental lattice parameter of 5.411 Å.[1] The bulk modulus, K_0 of 1.150 eV Å^{-3} is 184 GPa in standard units which compares to the experimentally measured values of 236 GPa[2] and 220 GPa.[3]

Surfaces were modelled using a slab model approach with a vacuum gap of 15 Å in the direction perpendicular to the slab. The (100) slab was modelled as a $p(2 \times 3)$ slab with 7 atomic layers (Figure S8*a*). The (110) and (111) surfaces were modelled as $p(2 \times 2)$ slabs consisting of 9 and 5 atomic layers, respectively (Figure S8*b* and *c*). This gives the surface vector dimensions $u = v = 15.470$ Å (111), dimensions $u = 10.939$ Å, $v = 15.470$ Å (110) and $u = v = 10.939$ Å (100). In addition, since the (100) surface has a nonzero dipole moment normal to the surface, the oxygen terminated surface was stabilised by removing half of the oxygen atoms on the top surface and adding them to the bottom of the slab. The large real space cell vectors allowed a lower *k*-point grid sampling to be used than was required for the bulk and so a *k*-point grid sampling of 3x3x1 was employed for all slab calculations.

The optimised CeO_2 surfaces were confirmed as minima by the absence of imaginary vibrational frequencies based on numerical finite difference vibrational mode calculations. The Hessian matrix was obtained by a finite difference of gradients with displacements of 0.001 Å.

The surface energies of each of the three surfaces of CeO_2 were calculated using:[4]

$$E_{surf}^{opt} = \frac{E_{slab} - nE_{bulk}}{S} - E_{surf}^{term} \quad \text{(S4)}$$

Where, E_{slab} is the energy of the optimised slab with the lower layers frozen at their bulk optimised co-ordinates, n is the ratio of the number of stoichiometric units in the slab and the bulk structure. S is the surface area of one of the surfaces created by making the slab, and E_{surf}^{term} is the surface energy calculated from the slab without relaxation to account for the fixing of the atoms in the lower face of the slab at their bulk positions. E_{surf}^{term} is obtained from a single point energy calculation on the freshly cleaved slab. In this case, the two surfaces are equivalent, therefore the term is obtained from a simpler equation:

$$E_{surf}^{term} = \frac{E_{slab}^{term} - nE_{bulk}}{2S} \quad \text{(S5)}$$

Surface energies reported in the literature utilizing the same PBE functionals with a similar slab are in good agreement with our values of 2.43 J m^{-2}, 1.75 J m^{-2}, 1.14 J m^{-2} and 2.05 J m^{-2}, 1.43 J m^{-2}, and 1.15 J m^{-2}, for both unrelaxed and relaxed surfaces of (100), (110), and (111) respectively. We used slabs that included seven atomic layers for (100) and (111), whereas five atomic layers was included for the (110) surfaces throughout the calculations. Three of the layers was set to relaxed while the subsurface layers beneath the relaxed layers were restricted to represent the bulk in all the CeO_2 surfaces.

Table S2. Calculated surface energies from slab model simulations.

Surface	E_{surf}^{term} / J m^{-2}	$E_{surf}^{term,b}$ / J m^{-2}	$E_{surf}^{term,a}$ / J m^{-2}	E_{surf}^{opt} / J m^{-2}	E_{surf}^{opt}(PW91)^{,a} / J m^{-2}	$E_{surf}^{opt}(Lit.)$ / J m^{-2}
(100)	2.43	2.37	---	2.06	---	1.57
(110)	1.75	1.55	1.25	1.43	1.05	1.35
(111)	1.15	1.06	0.69	1.14	0.68	1.04

Note: Lit: refers to reference *a*) PW91 values from reference 5.

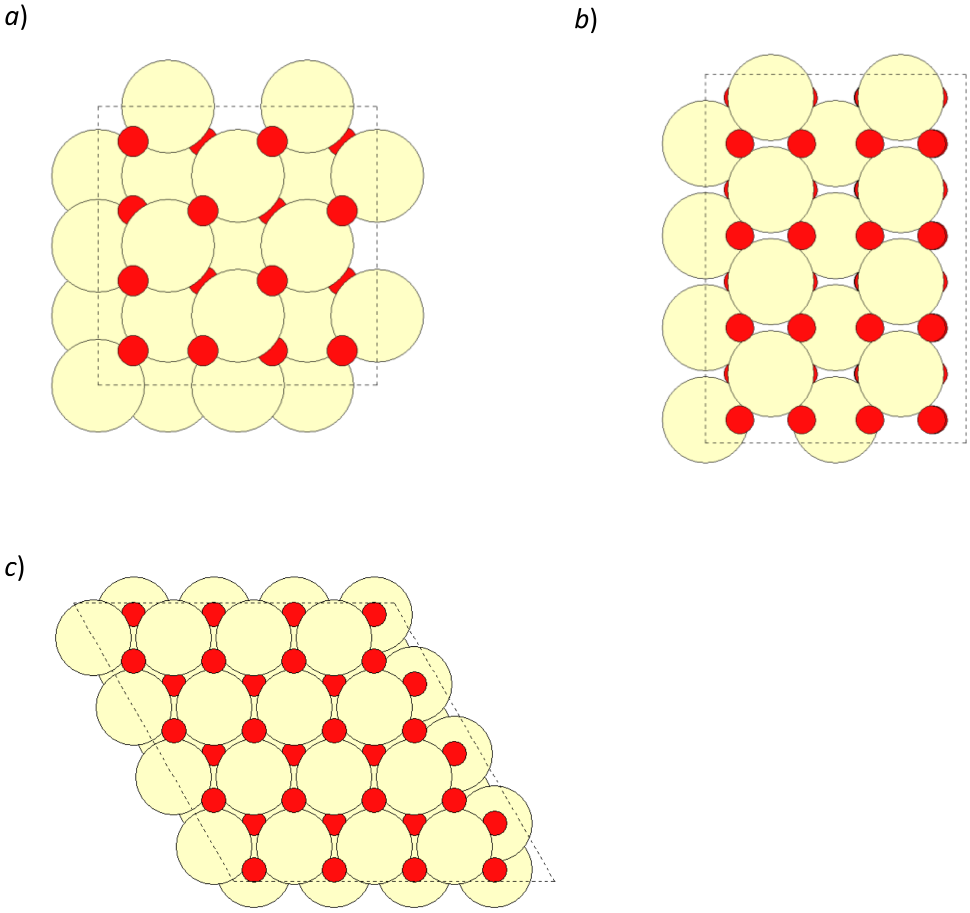

Figure S2. Plan views of the surfaces of slab models used for the simulation of *a*) CeO_2(100), *b*) CeO_2(110) and *c*) CeO_2(111). Images were produced using the ASE interface, atoms coloured: Ce; yellow, and O; red.

S2. Catalyst Characterisation

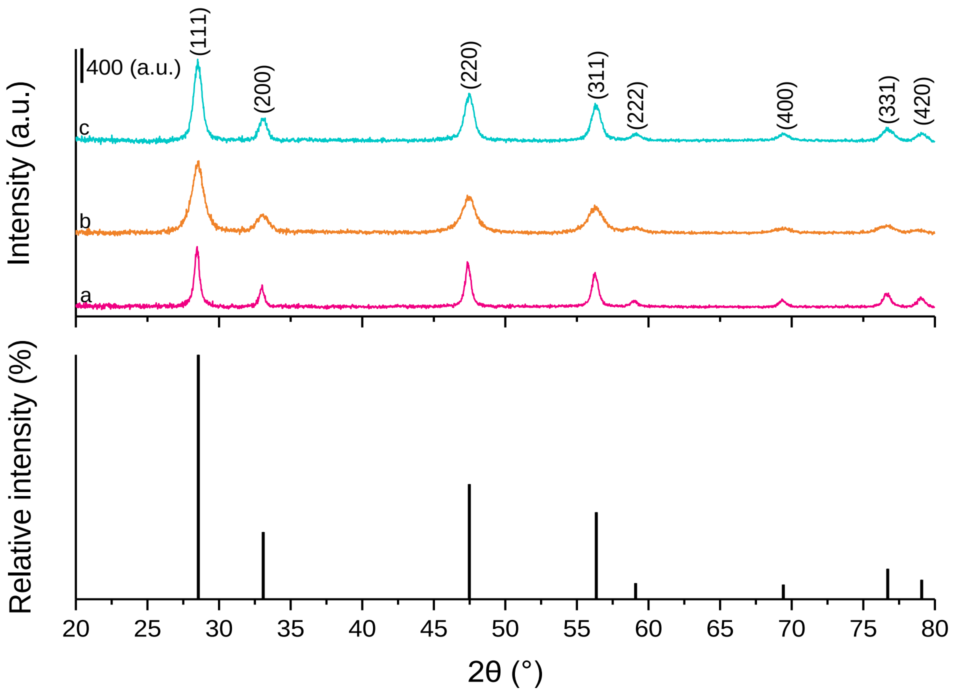

Figure S3. PXRD patterns of Figure S3. PXRD patterns of *a*) Ce-C, *b*) Ce-R, and *c*) Ce-P (stick patten for JCPDS 01-089-8436 shown for reference)

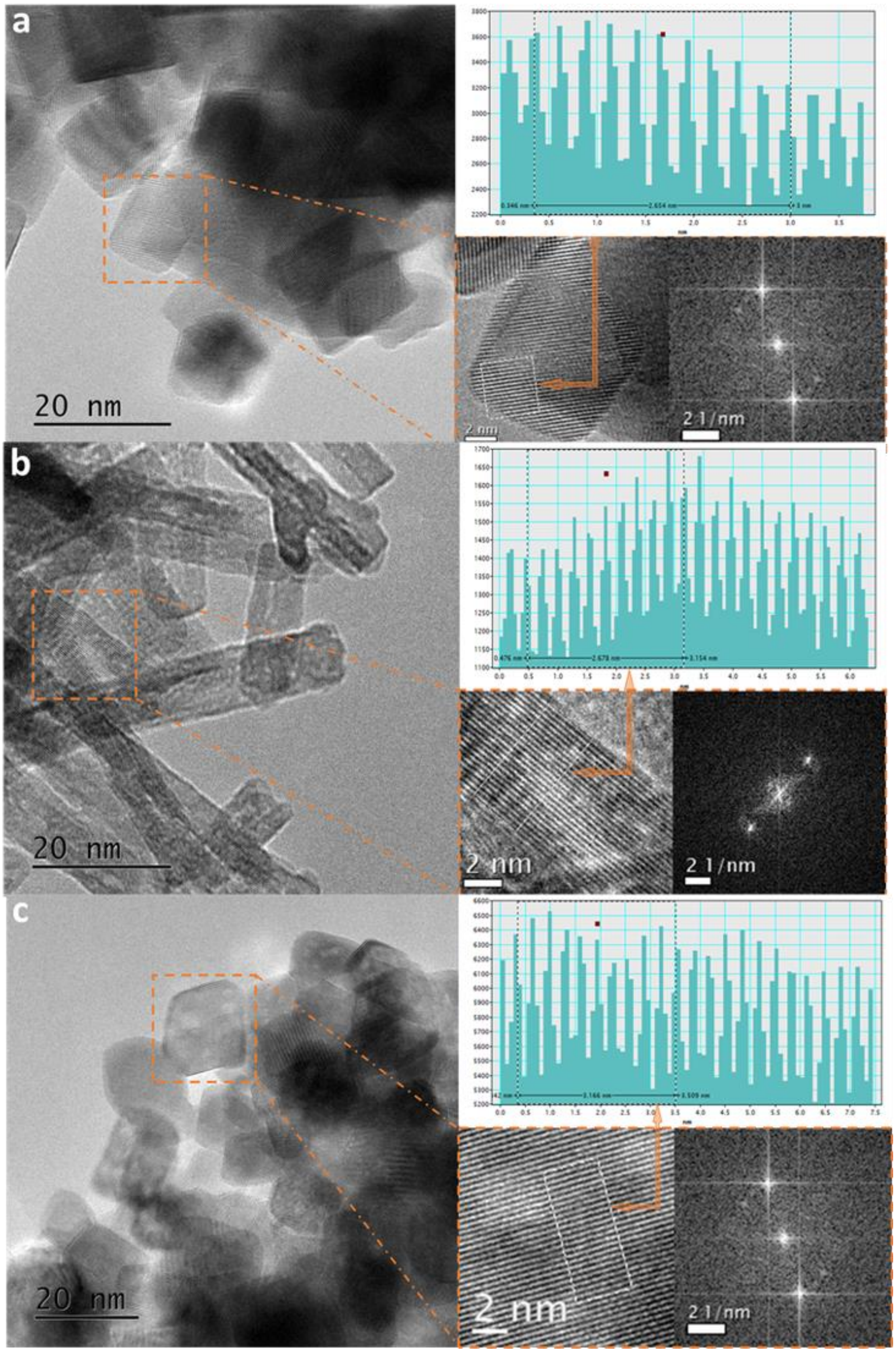

Figure S4. TEM images of *a*) Ce-C, *b*) Ce-R, and *c*) Ce-P showing the d-spacing and the corresponding FFT patterns

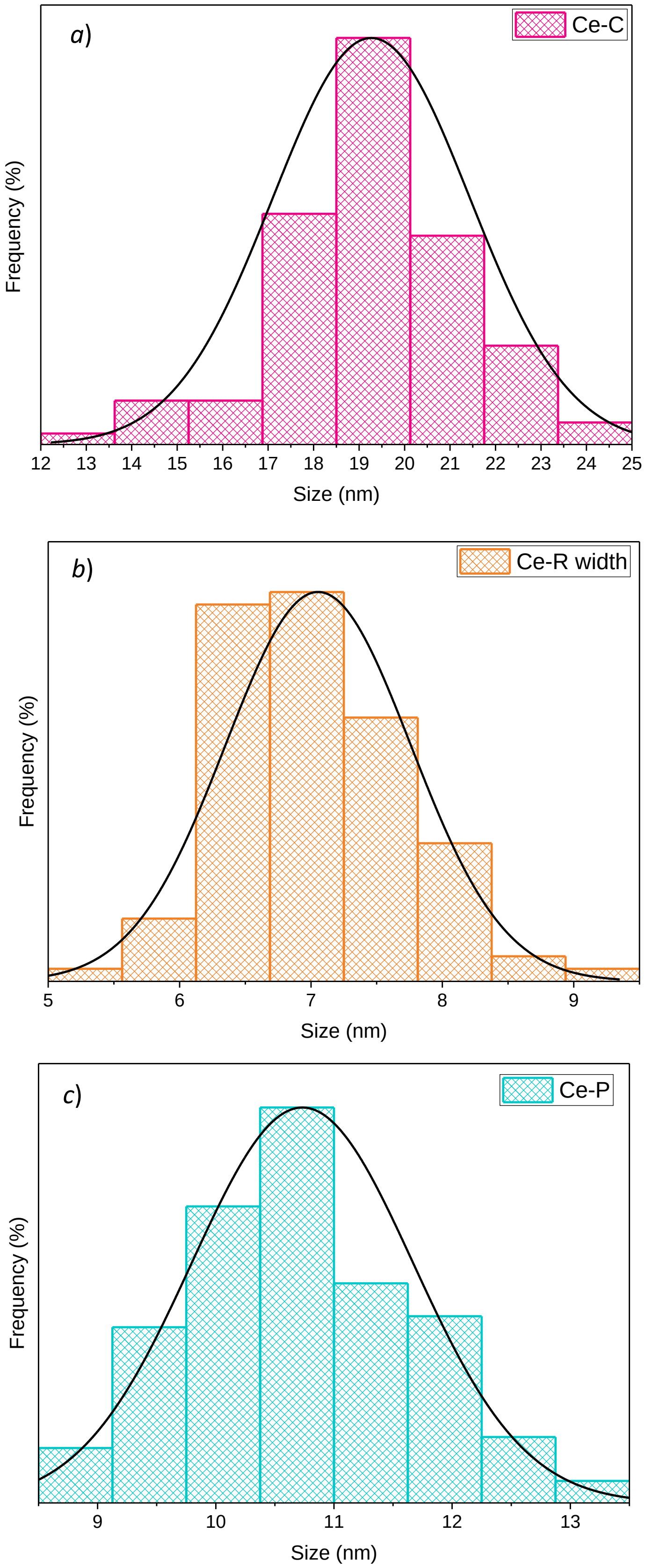

Figure S5. Particle size distribution for *a*) Ce-C, *b*) Ce-R, and *c*) Ce-P. Distributions were produced from measurements on 200-250 particles for Ce-C and Ce-P and 150 measurements for Ce-R. To assess the size of Ce-R particles the widths of the rods were taken, rods had a typical aspect ratio of 13.

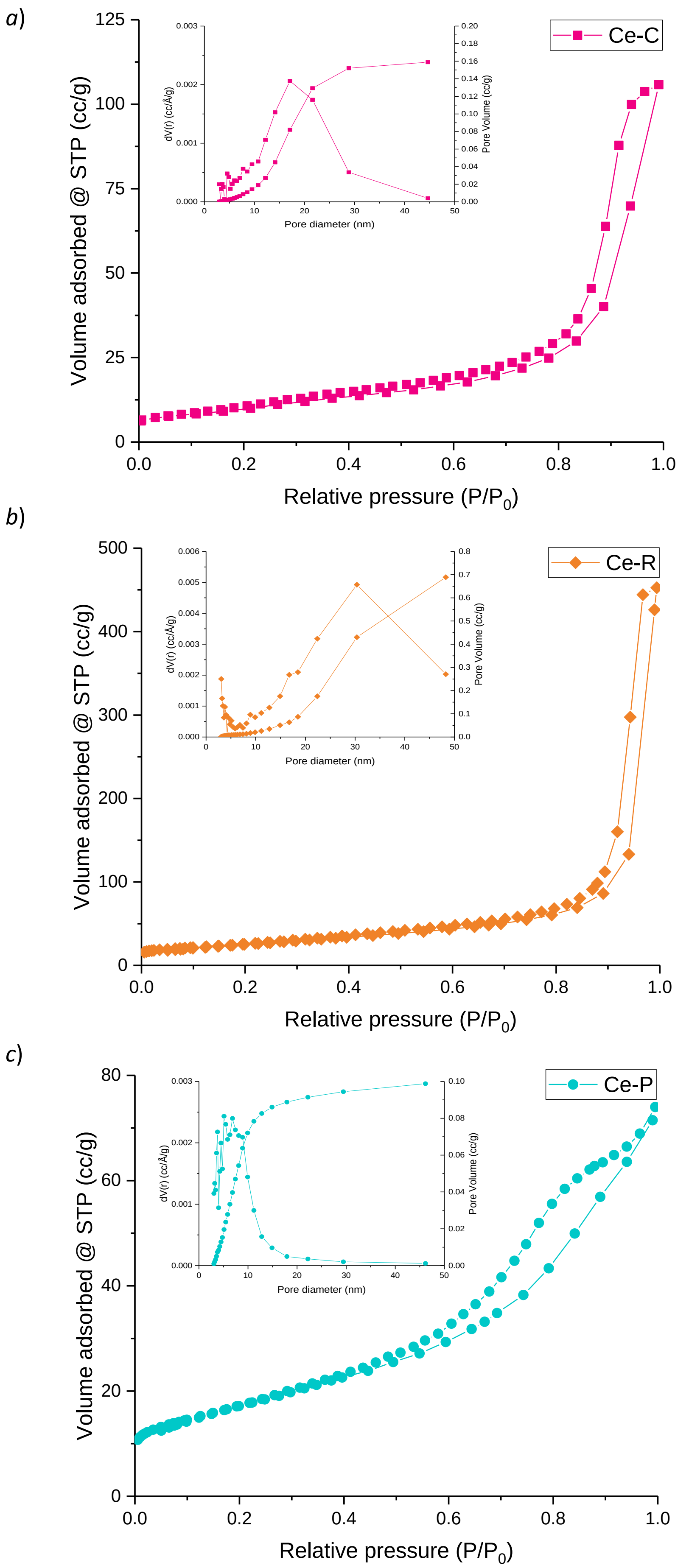

Figure S6. N_2 adsorption-desorption isotherms for *a*) Ce-C, *b*) Ce-R and *c*) Ce-P. In each case the inset shows the pore size distribution and cumulative pore volume calculated by the BJH method.

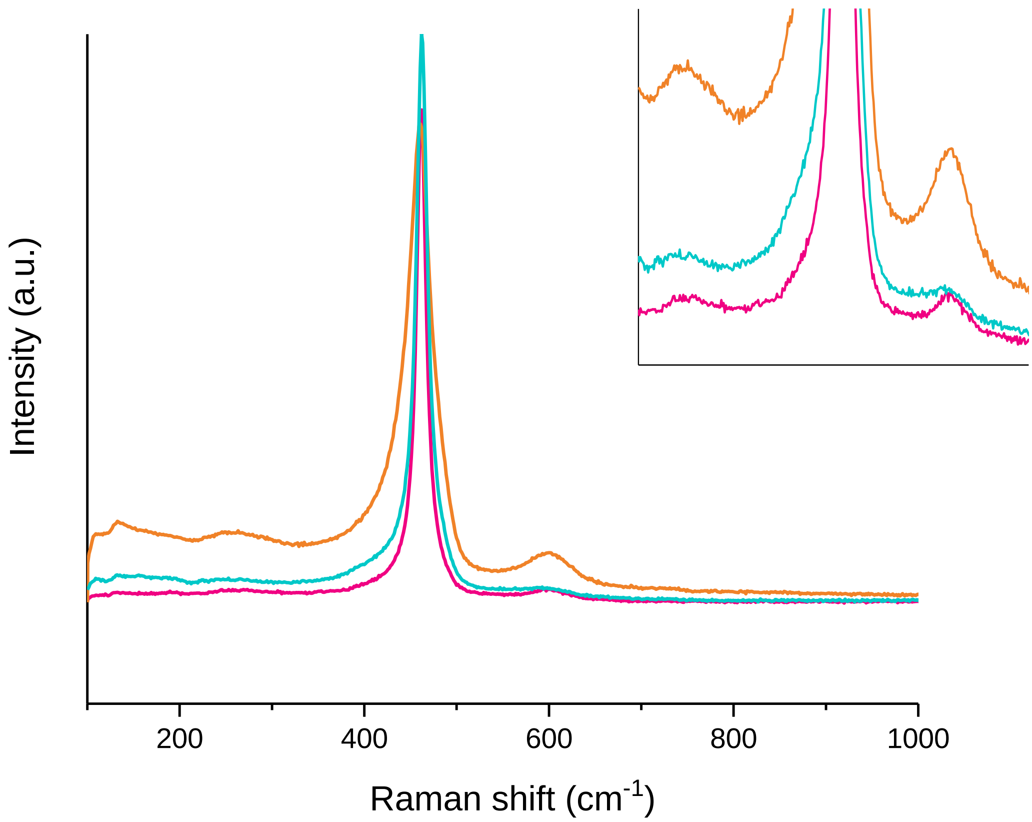

Figure S7. Visible Raman spectra of Ce-C (pink line), Ce-R (orange line) and polyhedra (blue line). Obtained at λ = 514 nm with a laser power of 1 %. Inset shows 200 – 700 cm^{-1} region.

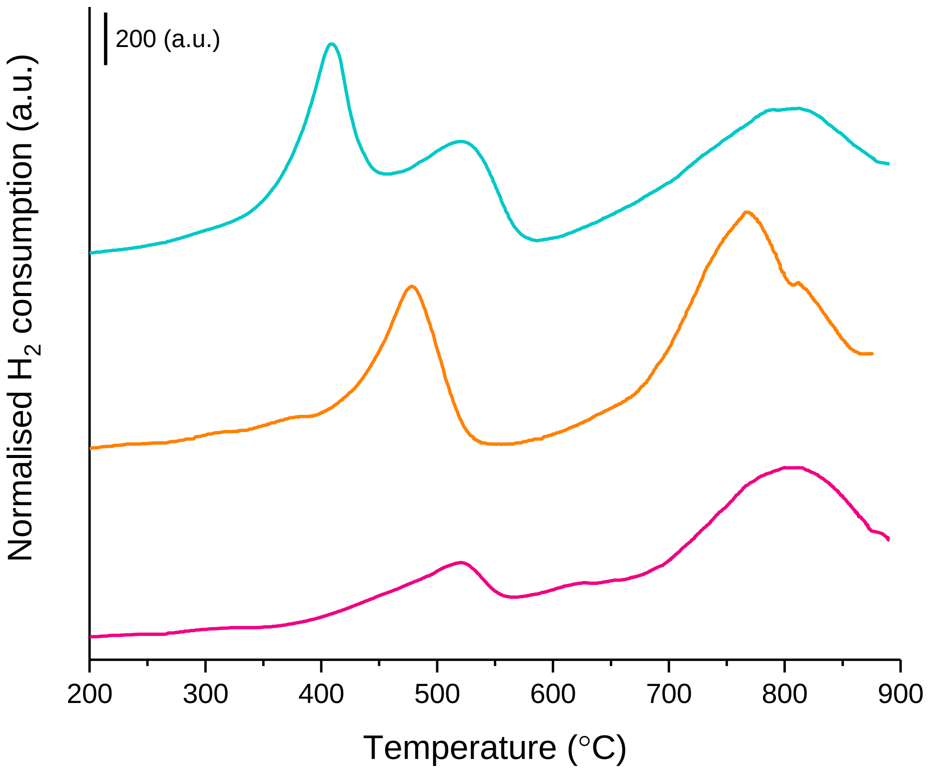

Figure S8. Temperature programmed reduction profiles using H_2 as reductant for Ce-C (pink), Ce-R (orange) and Ce-P (blue). All samples were heated at a rate of 10 K min^{-1} under 30 mL min^{-1} 10 % H_2/Ar.

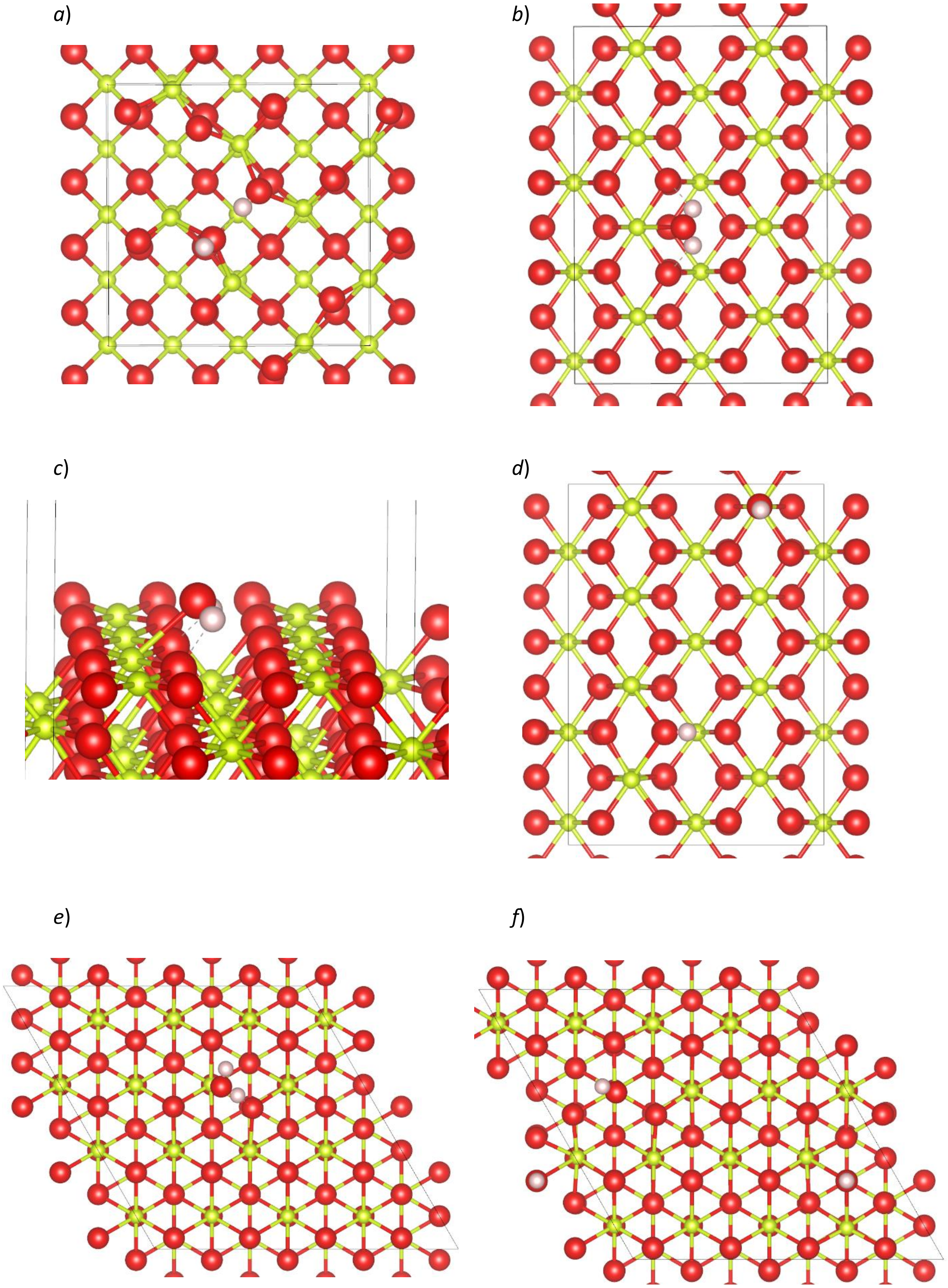

Figure S9. Structures for H_2O on ceria surfaces. *a*) Dissociated on CeO_2(100), *b*) and *c*) molecular adsorption on CeO_2(110), *d*) dissociated on CeO_2(110), *e*) molecular adsorption on CeO_2(111) and *f*) dissociated on CeO_2(111). Images produced using VESTA, atom colours, Ce; green, O; red and H; white.

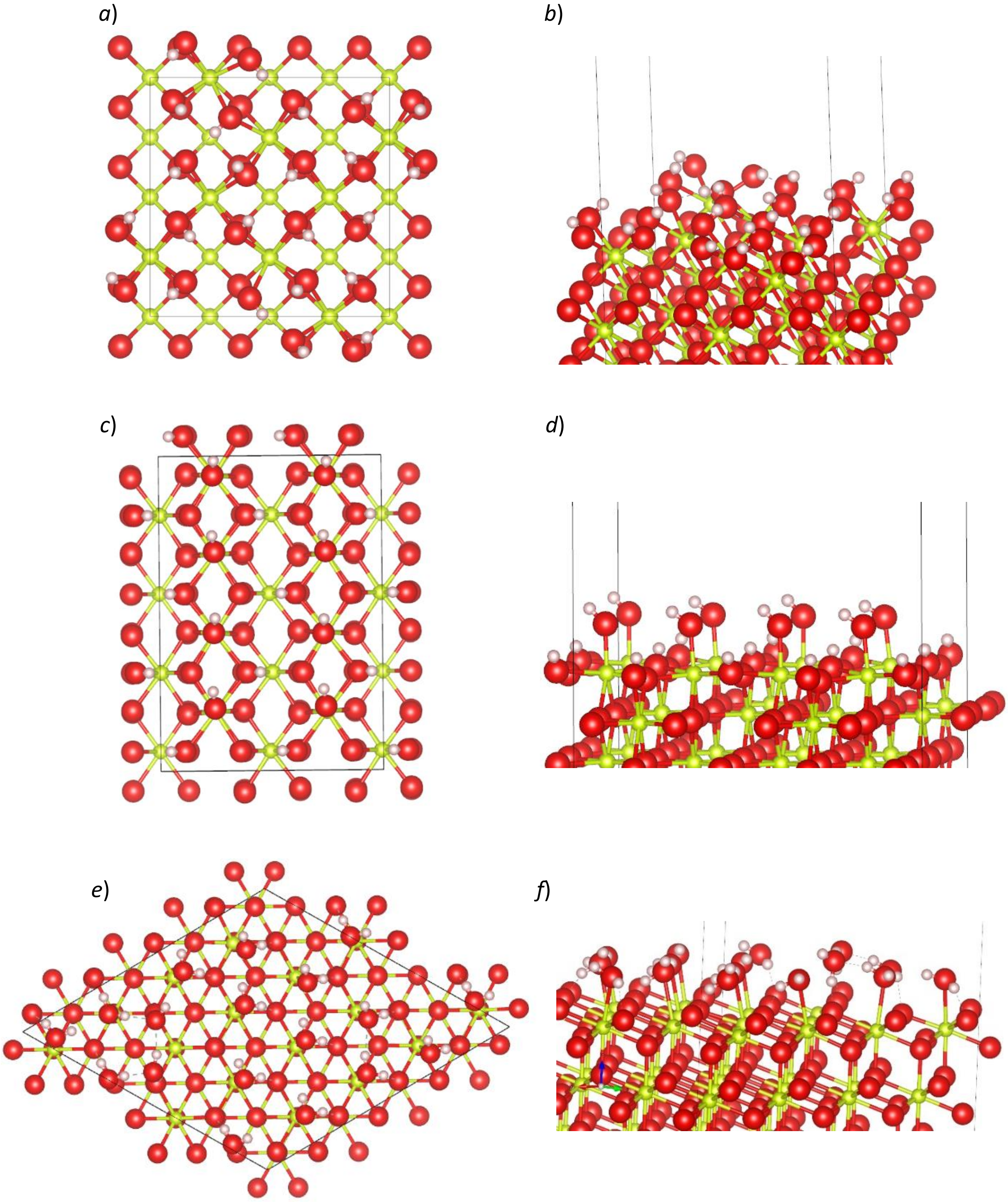

Figure S10. Optimised structures for 1 ML coverage of H_2O with dissociative adsorption on *a*) CeO_2(100), plan view, *b*) CeO_2(100), side view, *c*) CeO_2(110), plan view, *d*) CeO_2(110), side view and for molecular adsorption on *e*) CeO_2(111), plan view and *f*) CeO_2(111), side view. Images produced using VESTA, atom colours, Ce; green, O; red and H; white, molecular close contacts indicative of hydrogen bonding are shown as grey dotted lines.

S3 Thermochemistry calculations

To calculate the free energy of hydroxylation for the surfaces the VASP code was used to evaluate the vibrational modes of the relaxed clean surface, the surface with one monolayer coverage and an isolated water molecule. For vibrational calculations of slabs a single oxide layer and all adsorbate atoms were included in the degrees of freedom used to form the second derivative matrix. The enthalpy, *H*, entropy, *S*, and free energy, *G*, at a particular temperature, *T*, and pressure, *P*, are then calculated using the formulae:

$$H(T) = U_{elec} + ZPE + \int_0^T C_P dT \tag{S6}$$

$$S(T,P) = S_{trans} + S_{rot} + S_{vib} - k_B ln\left(\frac{P}{P^0}\right) \tag{S7}$$

$$G(T,P) = H(T) - TS(T,P) \tag{S8}$$

where U_{elec} is the PBE electronic energy of the system calculated by the VASP optimisation. The vibrational calculations provide the frequencies for the calculation of the zero point energy, ZPE and the heat capacity, C_p, and are used to calculate the vibrational contribution to the entropy, S_{vib}. For the slab calculations this is the only contribution but for the isolated water molecule the translational and rotational contributions to the entropy, S_{trans} and S_{rot}, are also estimated using standard statistical mechanics approaches. The required partial pressure of water in under the experimental reaction conditions was estimated as described in the following section.

For each system the calculations of the enthalpy and entropy was achieved using modules from the Atomic Simulation Environment python library.[6] As part of this work we have implemented python scripts to read the required data from VASP output files and carry out the set of calculations required to give the enthalpy, *H*, entropy, *S*, and free energy, *G*, change for the formation of the monolayer of water from the clean slab and isolated water molecules. The script makes additional checks such as ensuring that the number of degrees of freedom in reactant and product states is correctly matched.

S4 Estimation of the partial pressure of water under the experimental conditions

The partial pressure of water under experimental conditions (reaction temperature of 400 °C) was estimated using Dalton's law and the molar flow rates of glycerol, water, and argon carrier gas. The ideal gas law was used to calculate the mole fraction of water due to the large excess of inert carrier gas present.

	Glycerol partial pressure (mbar)	Water partial pressure (mbar)
CeO_2	0.47	0.60

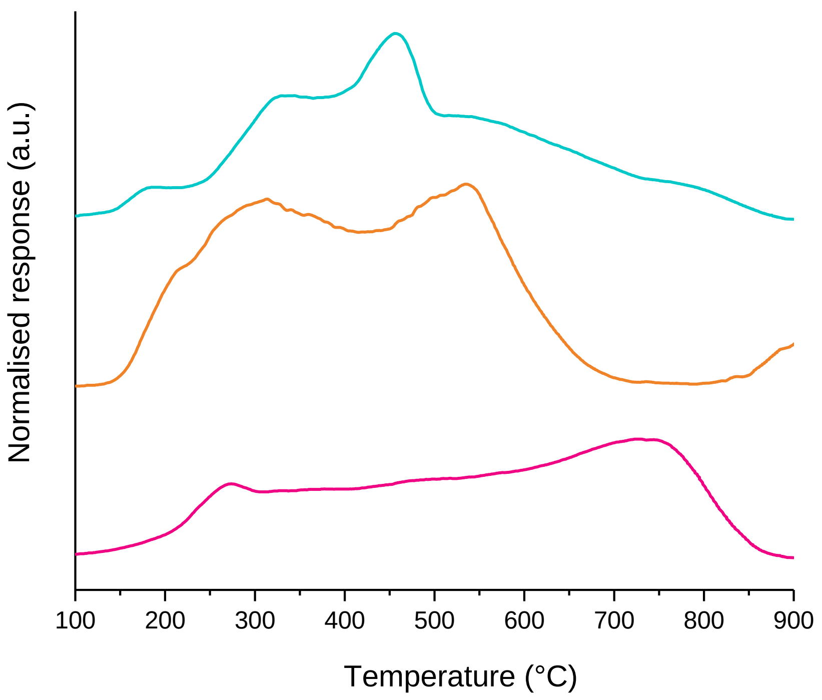

Figure S11. CO_2 temperature programmed desorption profiles for Ce-C (pink), Ce-R (orange) and Ce-P (blue). All samples were heated at a rate of 10 K min^{-1} under 30 mL min^{-1} He,

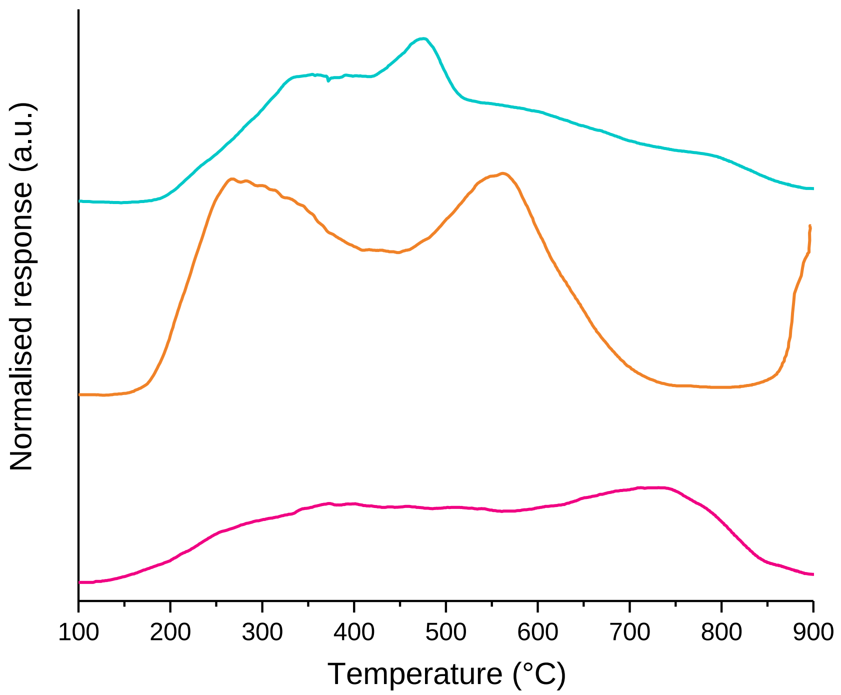

Figure S12. NH_3 temperature programmed desorption profiles for Ce-C (pink), Ce-R (orange) and Ce-P (blue). All samples were heated at a rate of 10 K min^{-1} under 30 mL min^{-1} He.

S5 Estimation of CO_2 desorption energy using the Redhead equation.

To estimate the desorption energy for CO_2 from the temperature of a TPD peak maximum, T_m, we employ the Redhead equation:[7]

$$\frac{E_{act}}{RT_m^2} = \frac{\nu_1}{\beta} exp\left(\frac{-E_{act}}{RT_m}\right) \tag{S9}$$

This equation is derived from the Polanyi-Wigner equation assuming coverage independent desorption and that the CO_2 adsorption/desorption process is first order. In equation (S9), R is the molar gas constant and ν_1 is the frequency factor in the Arrhenius expression for the desorption rate constant, which is taken to be $\nu_1 = 10^{13}$ s^{-1}. The parameter, β, is the heating rate of the TPD experiment, in our measurements β = 0.167 K s^{-1} throughout (Figure S10). To obtain the value of the activation energy, E_{act}, the right hand and left hand expressions in equation (S9) were evaluated for an initial guess value using an excel spreadsheet. The solver function was then used to minimise the square of the difference between the two sides of the equation by varying , E_{act}, for the values reported the difference between the left and right side was always less then 10^{-30} K^{-1}.

S6 Analysis of Reaction Products

Glycerol to MeOH: Full product list (Analysis conditions: GC1 Varian CP 3800 gas chromatograph equipped with a capillary column (ZB-Wax plus, 30 m x 0.53 mm x 1 µm). The injector port was maintained at 250 °C and a split-less injection was used. The initial column temperature (40 °C) was held for 2 mins, then ramped (20 °C min^{-1}) to 60 °C where it was held for 2 minutes before ramping to 220 °C (20 °C min^{-1}) and holding for 15 minutes. Products were analysed by an FID maintained at 300 °C. **GC2** Varian 450 gas chromatograph equipped with a capillary column (CP-Sil5CB, 50 m x 0.32 mm x 5 µm). The injector port was maintained at 200 °C and a 20:1 split ratio used. The initial column temperature (35 °C) was held for 15 mins and ramped (50 °C min^{-1}) to 100 °C where it was held for 3 minutes. Products were analysed by an FID with a methanizer, held at 200 °C and 350 °C respectively. **GC3** Varian CP3380 gas chromatograph equipped with a Porapak Q column. The injector was held at 50 °C; the column was maintained at 30 °C for 15 minutes. Products were analysed by a TCD, with the filament maintained at 200 °C.

Table S3. Retention times for full product list			
Product	Retention time / mins		
	GC1	GC2	GC3
Acetaldehyde	1.907		
Propionaldehyde	2.387		
Acetone	2.60		
Acrolein	2.840		
Butyraldehyde	3.133		
Methanol	3.48		
2-propanol	3.907		
Ethanol	4.013		
2,3-butanedione	4.547		
2-butanol	5.56		
1-propanol	5.800		
3-hexanone	5.907		
2-hexanone	6.360		
2-methyl-1-propanol	6.680		
Allyl alcohol	7.053		
Cyclopentanone	8.013		
Hydroxyacetone	9.373		
3-ethoxy-1-propanol	9.987		
Acetic acid	10.547		
Glycidol	10.790		
Propionic acid	11.240		
1,2-propanediol	11.747		
Ethylene glycol	12.013		
1,3-propanediol	13.080		
Phenol	14.760		
Glycerol	18.787		
CO		5.02	
CH_4		5.12	
CO_2		5.38	
H_2			2.222
O_2			2.902

Glycerol to MeOH: Qualitative analysis

Some additional qualitative analysis of the post reaction effluent was conducted by liquid chromatography-mass spectrometry (LCMS). This was conducted on a Bruker Amazon SL ion trap mass spectrometer which was operated in positive electrospray ion mode and coupled to a Thermo Ultimate HPLC system. The HPLC was equipped with a C-18 column (maintained at 40 ^{o}C) and utilized a gradient elution consisting of 0.1% formic acid in H_2O (A) and 0.1% formic acid in acetonitrile. 10 µL of sample was injected and the gradient elution was performed as illustrated in Table S3.

Table S4. The makeup of the mobile phase for the gradient elution.

Time / min	A / %	B / %
0.0	98	2
1.0	98	2
15.0	2	98
17.0	2	98
18.0	98	2
20.0	98	2

Note: A = 0.1 % formic acid in H_2O and B = 0.1 % formic acid in acetonitrile.

**Table S5a. Full product distribution at a space velocity of 3600 h^{-1} and reaction temperature of 320 °C**			
	**Ce-C**	**Ce-R**	**Ce-P**
**Glycerol conversion %**	17	65	58
**Product**	**Selectivity (%)**		
acetaldehyde	4.22	4.52	5.38
propionaldehyde	0.72	0.88	0.90
acetone	0.35	0.71	0.80
acrolein	14.18	2.36	1.84
butyraldehyde	0.00	0.07	0.08
methanol	**3.45**	**7.22**	**6.99**
2-propanol	0.07	0.06	0.00
ethanol	0.92	0.56	0.61
2,3-butanedione	0.51	2.22	1.96
2-butanol	0.00	0.01	0.09
1-propanol	0.33	0.15	0.12
3-hexanone	0.09	0.46	0.43
2-hexanone	0.03	0.02	0.00
2-methyl-1-propanol	0.06	0.03	0.00
allyl alcohol	1.15	0.97	1.29
cyclopentanone	0.15	0.19	0.16
hydroxyacetone	14.09	29.76	30.22
3-ethoxy-1-propanol	0.72	1.86	0.24
acetic acid	1.05	3.22	3.13
propionic acid	3.19	7.46	4.67
1,2-propanediol	2.40	7.12	6.00
unknown(s)	36.53	15.36	21.62
ethylene glycol	6.81	8.05	7.55
1,3-propanediol	2.64	0.46	0.34
phenol	0.00	0.00	0.00
CO	1.22	2.02	2.59
CH4	0.09	0.02	0.03
CO2	5.04	4.25	2.96

Table S5b. Full product distribution at a space velocity of 3600 h^{-1} and reaction temperature of 360 °C			
	**Ce-C**	**Ce-R**	**Ce-P**
**Glycerol conversion %**	29	98	96
**Product**	**Selectivity (%)**		
acetaldehyde	6.87	6.89	9.89
propionaldehyde	1.64	1.61	2.73
acetone	0.75	2.20	3.76
acrolein	12.68	1.87	1.49
butyraldehyde	0.00	0.14	0.21
methanol	**3.83**	**8.26**	**9.67**
2-propanol	0.08	0.19	0.17
ethanol	0.83	0.92	1.54
2,3-butanedione	1.08	2.35	1.06
2-butanol	0.05	0.00	0.15
1-propanol	0.32	0.33	0.43
3-hexanone	0.18	0.47	0.14
2-hexanone	0.03	0.04	0.05
2-methyl-1-propanol	0.05	0.04	0.04
allyl alcohol	2.37	1.38	2.17
cyclopentanone	0.30	0.49	0.68
hydroxyacetone	21.85	21.11	7.28
3-ethoxy-1-propanol	1.29	0.40	0.29
acetic acid	1.43	3.71	4.16
propionic acid	3.20	8.27	9.52
1,2-propanediol	2.70	5.96	7.86
unknown(s)	18.04	15.81	17.66
ethylene glycol	10.16	6.38	6.45
1,3-propanediol	1.38	0.22	0.00
phenol	0.00	0.21	0.00
CO	1.58	3.32	4.72
CH4	0.12	0.03	0.04
CO2	4.99	5.15	5.53

**Table S5c. Full product distribution at a space velocity of 3600 h^{-1} and reaction temperature of 400 °C**			
	**Ce-C**	**Ce-R**	**Ce-P**
**Glycerol conversion %**	91	> 99	> 99
**Product**	**Selectivity (%)**		
acetaldehyde	8.19	5.82	1.58
propionaldehyde	1.34	5.62	2.63
acetone	1.21	11.15	13.85
acrolein	1.07	0.77	0.29
butyraldehyde	0.12	0.37	0.24
methanol	**10.01**	**22.59**	**25.45**
2-propanol	0.10	1.59	1.73
ethanol	1.59	2.30	3.07
2,3-butanedione	1.02	3.65	5.50
2-butanol	0.01	0.03	0.17
1-propanol	0.38	0.98	1.63
3-hexanone	0.23	0.27	0.42
2-hexanone	0.06	0.41	0.68
2-methyl-1-propanol	0.03	0.08	0.23
allyl alcohol	1.68	2.71	1.35
cyclopentanone	1.03	1.59	1.63
hydroxyacetone	8.03	0.35	0.29
3-ethoxy-1-propanol	2.10	1.04	0.77
acetic acid	1.85	0.31	0.22
propionic acid	5.96	1.94	2.85
1,2-propanediol	5.46	0.34	0.15
unknown(s)	27.90	10.54	6.49
ethylene glycol	4.60	0.60	0.25
1,3-propanediol	1.62	0.15	0.12
phenol	0.51	0.29	0.19
CO	7.30	8.32	10.20
CH4	0.07	0.16	0.11
CO2	6.54	16.05	17.88

**Table S6a. Full product distribution at *iso*-conversion at C_{gly} ≈ 15% and a reaction temperature of 320 °C. Space velocities: Ce-C = 3600 h^{-1}, Ce-R = 11250 h^{-1}, Ce-P = 9000 h^{-1}.**			
	**Ce-C**	**Ce-R**	**Ce-P**
**Glycerol conversion %**	17	14	16
**Product**	**Selectivity (%)**		
acetaldehyde	4.22	4.77	5.21
propionaldehyde	0.72	0.97	0.56
acetone	0.35	0.55	0.52
acrolein	**14.18**	**3.21**	**1.52**
butyraldehyde	0.00	0.00	0.00
methanol	**3.46**	**7.69**	**8.35**
2-propanol	0.07	0.06	0.04
ethanol	0.92	1.08	0.28
2,3-butanedione	0.51	1.36	1.27
2-butanol	0.00	0.00	0.00
1-propanol	0.33	0.53	0.09
3-hexanone	0.09	0.34	0.20
2-hexanone	0.03	0.00	0.00
2-methyl-1-propanol	0.06	0.13	0.02
allyl alcohol	1.15	1.41	0.69
cyclopentanone	0.15	0.27	0.12
hydroxyacetone	**14.09**	**37.38**	**43.27**
3-ethoxy-1-propanol	0.72	0.92	0.12
acetic acid	1.05	1.29	2.15
propionic acid	3.19	2.27	3.10
1,2-propanediol	2.40	7.54	5.26
unknown(s)	36.53	16.15	16.38
ethylene glycol	6.81	6.97	7.29
1,3-propanediol	2.64	0.60	0.26
phenol	1.22	0.83	1.49
CO	0.09	0.00	0.00
CH4	5.04	3.47	1.63
CO2	1.15	1.41	0.09

**Table S6b. Full product distribution at *iso*-conversion at C_{gly} ≈ 99% and a reaction temperature of 400 °C. Space velocities: Ce-C = 1800 h^{-1}, Ce-R = 3600 h^{-1}, Ce-P = 3600 h^{-1}.**			
	**Ce-C**	**Ce-R**	**Ce-P**
**Glycerol conversion %**	> 99	> 99	> 99
**Product**	**Selectivity (%)**		
acetaldehyde	16.48	5.82	1.58
propionaldehyde	4.14	5.62	2.63
acetone	4.12	11.15	13.85
acrolein	2.74	0.77	0.29
butyraldehyde	0.22	0.37	0.24
methanol	**13.08**	**22.59**	**25.45**
2-propanol	0.41	1.59	1.73
ethanol	1.31	2.30	3.07
2,3-butanedione	1.79	3.65	5.50
2-butanol	0.22	0.03	0.17
1-propanol	0.00	0.98	1.63
3-hexanone	0.40	0.27	0.42
2-hexanone	0.06	0.41	0.68
2-methyl-1-propanol	0.02	0.08	0.23
allyl alcohol	4.09	2.71	1.35
cyclopentanone	0.45	1.59	1.63
hydroxyacetone	2.76	0.35	0.29
3-ethoxy-1-propanol	1.39	1.04	0.77
acetic acid	2.14	0.31	0.22
propionic acid	4.15	1.94	2.85
1,2-propanediol	1.42	0.34	0.15
unknown(s)	27.30	10.54	6.49
ethylene glycol	0.81	0.60	0.25
1,3-propanediol	1.05	0.15	0.12
phenol	0.80	0.29	0.19
CO	3.47	8.32	10.20
CH4	0.09	0.16	0.11
CO2	5.07	16.05	17.88

Table S7. Full product distribution at *iso*-conversion at C_{gly} ≈ 99% and a reaction temperature of 400 °C. Space velocities: Ce-C = 1800 h^{-1}, Ce-R = 3600 h^{-1}, Ce-P = 3600 h^{-1}.

Carbon component	Ce-C	Ce-R	Ce-P
**Coking**	1.8	2.4	0.8
**CO_x**	7.9	17.2	19.5
**CHN analysis**	84.8	75.7	76.1
**Total Carbon Content**	94.5	95.3	96.4

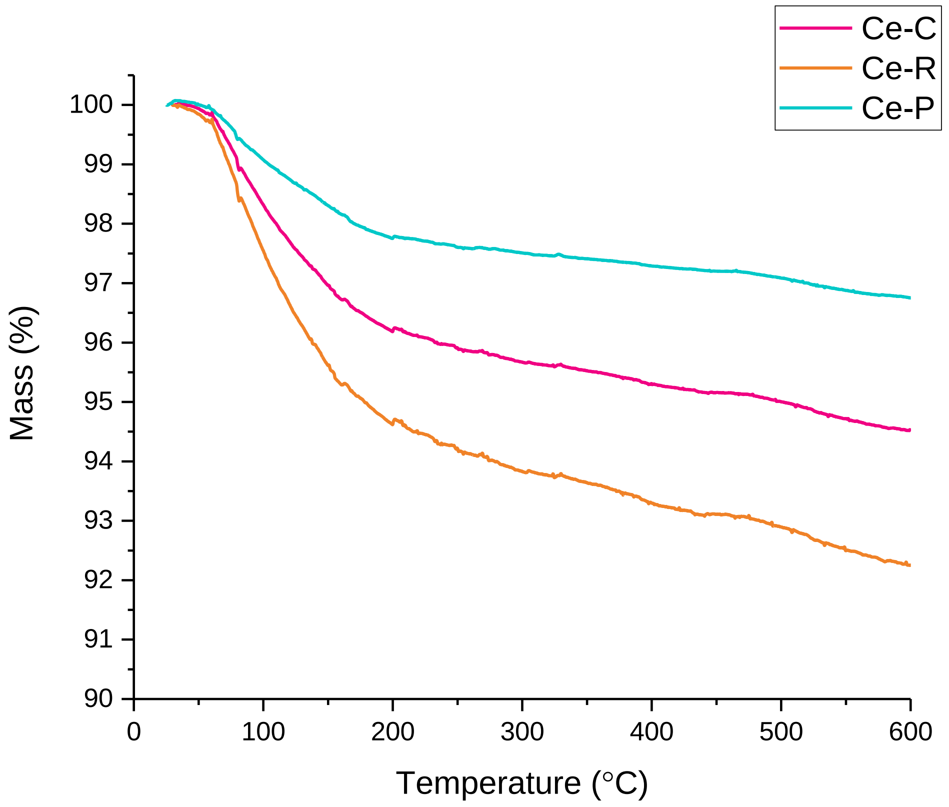

Figure S13. Post-reaction TGA of Ce-C (pink line), Ce-R (orange line) and Ce-P (blue line). Obtained under flowing air with a ramp rate of 5 °C min^{-1}.

Table S8. Glycerol conversion and main product selectivity as a function of contact time at various temperatures

Catalyst	T / °C	Space velocity / h^{-1}	Contact time /s	Conversion /%	Acrolein /%	MeOH /%	Hydroxyacetone /%
**Ce-C**	320	3600	1.0	15	14.2	3.5	14.1
**Ce-R**	320	11250	0.3	15	3.2	7.7	37.4
**Ce-P**	320	9000	0.4	15	1.5	8.3	43.3
**Ce-R**	320	3600	1.0	65	2.4	7.2	29.8
**Ce-P**	320	3600	1.0	58	1.8	7.0	30.2
**Ce-R**	320	1500	2.4	99	1.3	7.3	15.0
**Ce-P**	320	1800	2.0	91	1.5	6.5	15.5
**Ce-C**	360	3600	1.0	29	12.7	6.0	21.8
**Ce-R**	360	3600	1.0	98	1.9	10.5	21.1
**Ce-P**	360	3600	1.0	96	1.5	12.0	7.3
**Ce-R**	360	1500	2.4	100	0.3	13.1	2.3
**Ce-P**	360	1800	2.0	>99	0.5	9.4	2.0
**Ce-C**	400	1800	2.0	>99	2.7	13.1	2.8
**Ce-C**	400	3600	1.0	91	1.1	10.0	8.0
**Ce-R**	400	3600	1.0	>99	0.8	22.6	0.4
**Ce-P**	400	3600	1.0	>99	0.3	25.5	0.3

Table S9. Glycerol conversion to MeOH over various catalysts									
**Catalyst**	**Reaction T (°C)**	**Pressure (bar)**	**Glycerol wt.%**	**Glycerol flow rate (mL h^{-1})**	**Glycerol conversion (%)**	**Desired product**	**MeOH STY (g_{MeOH} g^{-1}_{cat} h^{-1})**	**MeOH selectivity (%)**	**Reference**
**Ce-P**	400	atmospheric	50	1.02	> 99	MeOH	201	25	This work
**CeO_2**	340	atmospheric	50	1.02	40	MeOH	25	34	[8]
**CeO_2**	315	atmospheric	36.2	50[a]	100	Acrolein	2235	8	[9]
**Ni/SiO_2**	320	60 (H_2)	100[b]	3	96	Mono-alcohols	3	5.5	[10]
**W/Al_2O_3**	320	60 (H_2)	100[b]	10	73	Mono-alcohols	91	41	[11]
**Supercritical water**	450	450	0.001	-[d]	10	-	No catalyst	3.6 (yield)	[12]
**CoZnO-ZIF**	210	20 (H_2)	40	1.2	99	EtOH	1450 $g_{ethanol}$ g^{-1}_{cat} h^{-1}	58 (ethanol)	[13]
**20CsZSM-5(1500)**	350	atmospheric	10	1.0	100	EtOH	51 $g_{ethanol}$ g^{-1}_{cat} h^{-1}	99.6 (ethanol)	[14]
a) corresponds to 0.85 mL/min glycerol flow; b) not reported – assumed; c) corresponds to 6.6 mL/min glycerol flow; d) not reported									

References

1. Eyring, L., Chapter 27 The Binary Rare Earth Oxides. In *Handbook on the Physics and Chemistry of Rare Earths*, Elsevier: 1979; Vol. 3, pp 337-399.
2. Gerward, L.; Olsen, J. S., Powder Diffraction Analysis of Cerium Dioxide at High Pressure. *Powder Diffraction* **1993,** *8*, 127-129.
3. Gerward, L.; Staun Olsen, J.; Petit, L.; Vaitheeswaran, G.; Kanchana, V.; Svane, A., Bulk Modulus of CeO2 and PrO2—An Experimental and Theoretical sStudy. *Journal of Alloys and Compounds* **2005,** *400*, 56-61.
4. Howard, K. L.; Willock, D. J., A Periodic DFT Study of the Activation of O2 by Au Nanoparticles on α-Fe2O3. *Faraday Discussions* **2011,** *152*, 135-151.
5. Skorodumova, N. V.; Baudin, M.; Hermansson, K., Surface Properties of CeO_2 from First Principles. *Physical Review B* **2004,** *69*, 075401.
6. Hjorth Larsen, A.; Jørgen Mortensen, J.; Blomqvist, J.; Castelli, I. E.; Christensen, R.; Dułak, M.; Friis, J.; Groves, M. N.; Hammer, B.; Hargus, C.; Hermes, E. D.; Jennings, P. C.; Bjerre Jensen, P.; Kermode, J.; Kitchin, J. R.; Leonhard Kolsbjerg, E.; Kubal, J.; Kaasbjerg, K.; Lysgaard, S.; Bergmann Maronsson, J.; Maxson, T.; Olsen, T.; Pastewka, L.; Peterson, A.; Rostgaard, C.; Schiøtz, J.; Schütt, O.; Strange, M.; Thygesen, K. S.; Vegge, T.; Vilhelmsen, L.; Walter, M.; Zeng, Z.; Jacobsen, K. W., The Atomic Simulation Environment-a Python Library for Working with Atoms. *Journal of physics. Condensed matter : an Institute of Physics journal* **2017,** *29*, 273002.
7. de Jong, A. M.; Niemantsverdriet, J. W., Thermal Desorption Analysis: Comparative Test of Ten Commonly Applied Procedures. *Surface Science* **1990,** *233*, 355-365.
8. Haider, M. H.; Dummer, N. F.; Knight, D. W.; Jenkins, R. L.; Howard, M.; Moulijn, J.; Taylor, S. H.; Hutchings, G. J., Efficient Green Methanol Synthesis from Glycerol. *Nature Chemistry* **2015,** *7*, 1028.
9. Chai, S.-H.; Wang, H.-P.; Liang, Y.; Xu, B.-Q., Sustainable Production of Acrolein: Investigation of Solid Acid-Base Catalysts for Gas-Phase Dehydration of Glycerol. *Green Chemistry* **2007,** *9*, 1130-1136.
10. van Ryneveld, E.; Mahomed, A. S.; van Heerden, P. S.; Green, M. J.; Friedrich, H. B., A Catalytic Route to Lower Alcohols from Glycerol using Ni-Supported Catalysts. *Green Chemistry* **2011,** *13*, 1819-1827.
11. Shozi, M. L.; Dasireddy, V. D. B. C.; Singh, S.; Mohlala, P.; Morgan, D. J.; Friedrich, H. B., Hydrogenolysis of Glycerol to Monoalcohols over Supported Mo and W Catalysts. *ACS Sustainable Chemistry & Engineering* **2016,** *4*, 5752-5760.
12. Bühler, W.; Dinjus, E.; Ederer, H. J.; Kruse, A.; Mas, C., Ionic Reactions and Pyrolysis of Glycerol as Competing Reaction Pathways in Near- and Supercritical Water. *The Journal of Supercritical Fluids* **2002,** *22*, 37-53.
13. Zhao, H.; Jiang, Y.; Chen, P.; Fu, J.; Lu, X.; Hou, Z., CoZn-ZIF-Derived ZnCo2O4-Framework for the Synthesis of Alcohols from Glycerol. *Green Chemistry* **2018,** *20*, 4299-4307.
14. Kostyniuk, A.; Bajec, D.; Likozar, B., One-Step Synthesis of Ethanol from Glycerol in a Gas Phase Packed Bed Reactor over Hierarchical Alkali-Treated Zeolite Catalyst Materials. *Green Chemistry* **2020,** *22*, 753-765.
